# A solid-phase enzymatic synthesis platform for the facile production of 2′-fluoroarabinonucleic acid (FANA) and chimeric XNA oligonucleotides using an evolved XNA polymerase

**DOI:** 10.1093/nar/gkaf567

**Published:** 2025-06-26

**Authors:** Binliang Zhang, Yuhui Du, Jingxing Zhang, Xingyun Ma, Yanjia Qin, Rui Tao, Minglan Luo, Jing Wu, Leping Sun, Gan Zhu, Hantao Luo, Junlin Wen, Chenghe Xiong, Hui Mei, Tingjian Chen

**Affiliations:** MOE International Joint Research Laboratory on Synthetic Biology and Medicines, School of Biology and Biological Engineering, South China University of Technology, Guangzhou 510006, China; MOE International Joint Research Laboratory on Synthetic Biology and Medicines, School of Biology and Biological Engineering, South China University of Technology, Guangzhou 510006, China; MOE International Joint Research Laboratory on Synthetic Biology and Medicines, School of Biology and Biological Engineering, South China University of Technology, Guangzhou 510006, China; MOE International Joint Research Laboratory on Synthetic Biology and Medicines, School of Biology and Biological Engineering, South China University of Technology, Guangzhou 510006, China; MOE International Joint Research Laboratory on Synthetic Biology and Medicines, School of Biology and Biological Engineering, South China University of Technology, Guangzhou 510006, China; MOE International Joint Research Laboratory on Synthetic Biology and Medicines, School of Biology and Biological Engineering, South China University of Technology, Guangzhou 510006, China; MOE International Joint Research Laboratory on Synthetic Biology and Medicines, School of Biology and Biological Engineering, South China University of Technology, Guangzhou 510006, China; MOE International Joint Research Laboratory on Synthetic Biology and Medicines, School of Biology and Biological Engineering, South China University of Technology, Guangzhou 510006, China; MOE International Joint Research Laboratory on Synthetic Biology and Medicines, School of Biology and Biological Engineering, South China University of Technology, Guangzhou 510006, China; MOE International Joint Research Laboratory on Synthetic Biology and Medicines, School of Biology and Biological Engineering, South China University of Technology, Guangzhou 510006, China; MOE International Joint Research Laboratory on Synthetic Biology and Medicines, School of Biology and Biological Engineering, South China University of Technology, Guangzhou 510006, China; Shenzhen Key Laboratory of Synthetic Genomics, Guangdong Provincial Key Laboratory of Synthetic Genomics, State Key Laboratory of Quantitative Synthetic Biology, Shenzhen Institute of Synthetic Biology, Shenzhen Institutes of Advanced Technology, Chinese Academy of Sciences, Shenzhen 518055, China; Shenzhen Key Laboratory of Synthetic Genomics, Guangdong Provincial Key Laboratory of Synthetic Genomics, State Key Laboratory of Quantitative Synthetic Biology, Shenzhen Institute of Synthetic Biology, Shenzhen Institutes of Advanced Technology, Chinese Academy of Sciences, Shenzhen 518055, China; Shenzhen Key Laboratory of Synthetic Genomics, Guangdong Provincial Key Laboratory of Synthetic Genomics, State Key Laboratory of Quantitative Synthetic Biology, Shenzhen Institute of Synthetic Biology, Shenzhen Institutes of Advanced Technology, Chinese Academy of Sciences, Shenzhen 518055, China; MOE International Joint Research Laboratory on Synthetic Biology and Medicines, School of Biology and Biological Engineering, South China University of Technology, Guangzhou 510006, China

## Abstract

Xenobiotic nucleic acids (XNAs) significantly expand the range of genetic polymers and serve as promising alternatives to DNA and RNA for numerous biological applications. However, the extensive exploration and application of XNAs are limited by low sustainability and yields in solid-phase oligonucleotide synthesis, as well as by the unavailability of efficient XNA polymerases for polymerase-mediated XNA production. To address the limitations in XNA production, we developed a solid-phase enzymatic XNA oligonucleotide synthesis platform using a laboratory-evolved XNA polymerase, SFM5-7, which exhibits excellent activity for synthesizing DNA, RNA, 2′-fluoroarabinonucleic acid (FANA), and other 2′-modified XNA oligonucleotides. This platform employs ribonucleotide insertion and alkaline cleavage of the oligonucleotide product before and after SFM5-7-mediated XNA synthesis, enabling recycled XNA synthesis through the reuse of a bead-immobilized self-priming hairpin DNA template. The platform’s potential and versatility are demonstrated by the production of FANA, 2′-modified RNAs, chimeric XNAs, 5′-end-labeled FANA, and an active FANAzyme. This platform should facilitate the customized production of functional XNAs with programmable modifications, accelerating their applications in biotechnology and biomedicine.

## Graphical Abstract

**Graphical Abstract:**
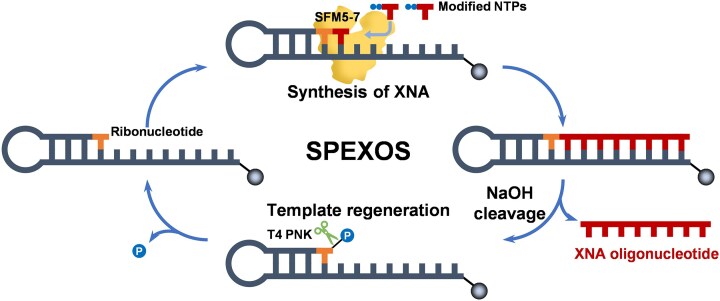

## Introduction

**Figure 1.**
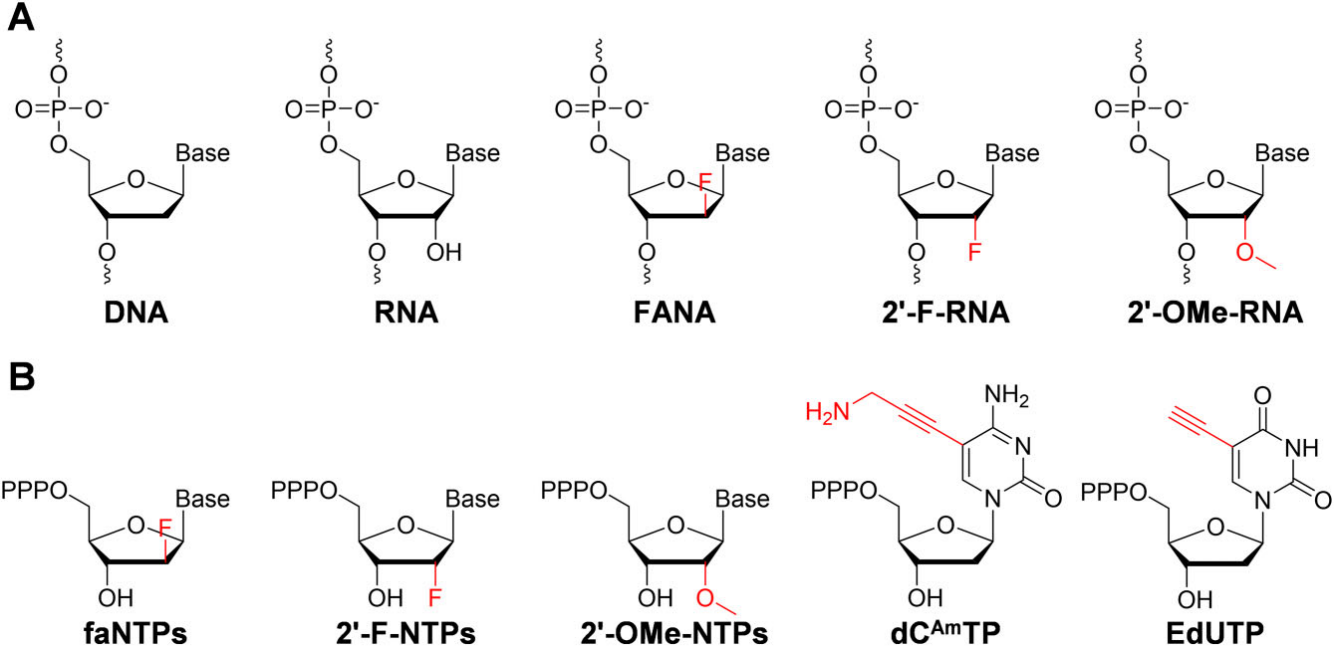
Chemical structures of the nucleic acids and modified nucleoside triphosphates involved in this study. (**A**) DNA, RNA, FANA, 2′-F-RNA, and 2′-OMe-RNA. (**B**) faNTPs, 2′-F-NTPs, 2′-OMe-NTPs, dC^Am^TP, and EdUTP.

Xenobiotic nucleic acids (XNAs) are a group of artificially synthesized genetic polymers analogous to DNA and RNA and possessing unnatural moieties in their structural units [1–3]. The unnatural moieties endow XNA molecules with intriguing properties such as increased stability and expanded functionality, making them irreplaceable tools for the development of a variety of modern nucleic acid-based technologies in biotechnology and biomedicine [4–7]. Among various XNAs, 2′-fluoroarabinonucleic acid (FANA) is one of the most studied and applied. It is a DNA analogue in which the 2′-deoxyribose is replaced by a 2′-deoxy-2′-fluoroarabinose (Fig. 1A) [8, 9]. Due to the structural feature, FANA possesses a variety of unique properties and is widely used in many fields, including biosensing, synthetic biology, biomedicine, and materials science [10–14]. For example, FANA-modified siRNAs have shown superior gene-silencing capability and enhanced stability in serum, making them attractive molecules for the development of RNA interference therapeutics [15, 16]. The ability of FANA to activate RNase H when hybridizing with RNA also makes it valuable in the design of antisense therapeutics [8, 17]. Recently, FANA aptamers with high affinity to the targets and FANAzymes capable of efficiently cleaving and ligating RNA have been reported, further expanding the scope of FANA’s functions [11, 18–24]. Notably, FANA has exhibited high resistance to acid hydrolysis and a wider operating pH range than those of DNA and RNA [25], which should allow the use and manipulation of FANA in more acidic or alkaline solutions without compromising its structural integrity.

To fully explore and utilize XNA in various fields, it is essential to develop efficient tool enzymes, particularly XNA polymerases that can catalyze efficient and accurate XNA synthesis. To date, reported efficient FANA polymerases are mainly wild-type or mutant family B DNA polymerases (DNAPs), including phi29 DNAP (D12A), Deep Vent DNAP (exo^−^), 9°N DNAP (exo^−^), Tgo-D4K (a mutant of Tgo DNAP), RT521 (a mutant of Tgo DNAP), and Kod-RSGA (a mutant of Kod DNAP) [2, 10, 11, 26, 27], while efficient FANA polymerases derived from family A DNAPs have barely been reported. Previously, employing directed protein evolution approach, the Stoffel fragment (SF) of Taq DNAP has been successfully engineered to be a mutant (SFM4-3) capable of efficiently and faithfully synthesizing, reverse transcribing, or even amplifying RNA and a range of 2′-modified nucleic acids, such as 2′-*O*-methyl (2′-OMe)-modified nucleic acids and 2′-fluoro (2′-F)-modified nucleic acids (Fig. 1A) [28–31]. Another evolved SF mutant, SFM4-6, exhibits good efficiency in synthesizing 2′-OMe-RNA, while mutant SFM4-9 effectively reverse transcribes 2′-OMe-RNA to DNA [28]. Further evolved mutant SFM5-7 can mediate the synthesis, reverse transcription, replication, and inter-transcription of DNA, RNA, 2′-OMe-RNA, and 2′-F-RNA, and the synthesis of 2′-OMe-RNA with bigger lengths is more efficient by SFM5-7 than by SFM4-3 and SFM4-6 [29]. Although these SF mutants have already demonstrated a significantly expanded substrate repertoire, their capability to synthesize FANA remains uninvestigated. Additionally, their ability to generate chimeric XNA oligonucleotides from mixed DNA, RNA, 2′-modified RNA, or FANA nucleoside triphosphates has not yet been systematically studied. Chimeric XNAs generated from combinations of different XNAs may enhance the chemical and structural diversity available for XNA aptamer or XNAzyme selection and functionality [32–34].

Efficient and facile production of XNA oligonucleotides is a prerequisite for the extensive application of XNAs. Traditionally, oligonucleotides, including many XNA or modified oligonucleotides, are produced via solid-phase chemical synthesis, which is powerful but with some intrinsic limitations, including unsatisfactory coupling efficiency, declining product yield and purity along with the increasing of product length, and massive use of reagents and solvents that are environmentally unfriendly [35–37]. To address these limitations, efforts have focused on developing enzymatic methods for producing XNA or modified oligonucleotides. This typically involves using an efficient polymerase for the synthesis of the XNA or modified oligonucleotides, with DNase I, endonuclease V from *Thermotoga neapolitana*, or a nicking endonuclease employed to remove the DNA primer and template from the XNA or modified oligonucleotide products [30, 38–42]. In another strategy, a primer containing a single 2′-deoxyuridine and cleavage by uracil-DNA glycosylase (UDG) have been used for the release of hypermodified RNA oligonucleotides after polymerase-mediated synthesis [43]. Despite significant progress, these enzymatic methods have several limitations, including the requirement for a specifically designed DNA template and the use of an additional enzyme (DNase I, endonuclease V, or a nicking endonuclease) for DNA digestion. Besides, DNase I’s inefficiency in fully degrading DNA primers or templates ligated or hybridized with certain XNA products can lead to impurities in the final XNA oligonucleotides. Furthermore, methods employing DNase I are unsuitable for producing XNA oligonucleotides containing nucleotides that are less tolerant to DNase I, such as FANA oligonucleotides [44, 45]. Thus, a novel and efficient XNA synthesis platform is urgently needed to address these limitations.

In this study, we developed a solid-phase enzymatic XNA oligonucleotide synthesis (SPEXOS) platform for the convenient, efficient, and sustainable production of XNA oligonucleotides using laboratory-evolved XNA polymerases. To this end, we first explored the activity of four laboratory-evolved SF variants, SFM4-3, SFM4-6, SFM4-9, and SFM5-7, for the DNA-templated synthesis of FANA with varying lengths. Then, the most efficient variant, SFM5-7, was further characterized by steady-state kinetic and sequencing experiments for its activity and fidelity for FANA synthesis. Taking advantage of the outstanding efficiency and reasonable fidelity of SFM5-7 for the synthesis of DNA, RNA, FANA, and other XNAs, we established the SPEXOS platform by incorporating ribonucleotide insertion and alkaline cleavage of the oligonucleotide product before and after SFM5-7-mediated XNA synthesis, thus avoiding the drawbacks of the use of DNase I, such as limited product purity and yield from digestion. The platform’s efficiency and versatility were demonstrated by producing various XNA oligonucleotides, including FANA, 2′-F-RNA, and 2′-OMe-RNA, as well as chimeric constructs like 2′-F-RNA/2′-OMe-RNA, FANA/DNA, FANA/2′-F-RNA, FANA/2′-F-RNA/2′-OMe-RNA, FANA/DNA/2′-OMe-RNA, and FANA/DNA/2′-F-RNA/2′-OMe-RNA. It also enabled the enzymatic production of FANA oligonucleotides with single and double 5′-end labels, useful for attaching functional groups in molecular diagnostics and bioimaging. Finally, a FANAzyme was prepared with this platform and its activity was verified, demonstrating the effectiveness of this platform in producing functional XNA oligonucleotides.

## Materials and methods

### Materials

Q5 DNA polymerase, EcoRI, HindIII, T4 polynucleotide kinase (PNK), 10× standard Taq reaction buffer, deoxyribonucleoside triphosphates (dNTPs), and ribonucleoside triphosphates (rNTPs) were purchased from New England Biolabs (Ipswich, MA, USA). Oligonucleotides, streptavidin (SA), Ni-NTA resin, and 2× TBE–urea sample buffer were purchased from Sangon (Shanghai, China). The chemically synthesized oligonucleotide of FANAzyme FR6_1_BRaf600 was purchased from Primerna NAT Co., Ltd (Shanghai, China). 5-Ethynyl-2′-deoxyuridine 5′-triphosphate (EdUTP), 5-propargylamino-2′-deoxycytidine 5′-triphosphate (dC^Am^TP), 2′-fluoro-2′-deoxyribonucleoside 5′-triphosphates (2′-F-NTPs), and 2′-*O*-methyl-ribonucleoside 5′-triphosphates (2′-OMe-NTPs) were purchased from Huaren (Wuhu, China). 2′-Deoxy-2′-fluoroarabinonucleoside 5′-triphosphates (faNTPs) were synthesized in the lab or purchased from Huaren (Wuhu, China). Zymo DNA Clean & Concentrator-5 and Zymo ssDNA/RNA Clean & Concentrator kits were purchased from Zymo Research (Irvine, CA, USA). RiboLock RNase inhibitor was purchased from Thermo Fisher Scientific (Waltham, MA, USA). Azide-AF488 and Cyber Gold nucleic acid stain were purchased from Biolife Biotech Inc. (Xi’an, China). A 2× SYBR Green qPCR premix was purchased from Xinkailai Biotechnology (Guangzhou, China). Dibenzocyclooctyne (DBCO)-coated magnetic beads were purchased from Bangs Laboratories (Fishers, IN, USA). SA-coated magnetic beads were purchased from BEAVER (Suzhou, China).

### Expression and purification of the proteins of SF WT and SF mutants

The wild-type (WT) and mutants of the Stoffel fragment (SF) of Taq DNA polymerase (DNAP) (SF WT, SFM4-3, SFM4-6, SFM4-9, and SFM5-7) were expressed and purified as previously described [28, 29]. Briefly, single colonies of *Escherichia coli* BL21(DE3)/pLysS cells harboring the expression plasmid for each of the DNAPs [pET23b (+)-SF WT, SFM4-3, SFM4-6, SFM4-9, or SFM5-7] were cultured in 2× YT medium containing 100 μg/ml ampicillin and 25 μg/ml chloramphenicol at 37°C with shaking overnight. The overnight cultures were, respectively, diluted at a ratio of 1:100 with fresh medium of the same composition and grown at 37°C to log phase (OD_600_ = 0.6–0.8) before the addition of 0.4 mM isopropylthio-β-galactoside (IPTG), followed by incubation of the culture at 25°C for 12 h. The cells were harvested by centrifugation at 3500 rpm for 0.5 h, resuspended in washing buffer A (50 mM Tris–HCl, 150 mM NaCl, and 5 mM imidazole, pH 8.0), and disrupted by high-pressure homogenization. Then, the cell lysates were incubated at 70°C for 15 min to precipitate the cellular proteins. After removal of the precipitated proteins by centrifugation at 10 000 rpm for 0.5 h, the proteins of the DNAPs in the supernatants were purified using Ni-NTA resin, concentrated with Amicon Ultra centrifugal filters (MWCO 50 kDa), and stored in 50% glycerol at −20°C before use.

### Activity assay of SF WT and SF mutants for the synthesis of FANA

5′-FAM-labeled DNA primer FAM-P20 was mixed with DNA template T50 or T70 (Supplementary Table S1) in standard Taq reaction buffer, incubated at 95°C for 10 min, and slowly cooled down to room temperature to anneal the primer to the template. To carry out the primer extension reaction for FANA synthesis, 100 nM primer/150 nM template was mixed with 0.4 mM each of faNTPs, 5 mM extra MgCl_2_, 0 or 1 mM MnCl_2_, and 2.5 μM SF WT or one of the SF mutants in 1× standard Taq reaction buffer. Then, the mixture was subjected to the following thermocycling program: 10 cycles of (50°C, 1 h; 70°C, 10 min); 50°C, 1 h. The reactions were quenched by the addition of 2× TBE–urea sample buffer and the resultant solutions were incubated at 95°C for 10 min, and the products were analyzed with 15% denaturing PAGE (polyacrylamide gel electrophoresis) gels containing 8 M urea. The full-length product rates were calculated from the band intensities of the products quantified from the gel images. All experiments were triplicated.

### Steady-state kinetic experiments for the incorporation of faNTPs by SF WT and SFM5-7

5′-Cy3-labeled primer Cy3-P22 was mixed with DNA template T-A, T-U, T-C, or T-G (Supplementary Table S1) in standard Taq reaction buffer, incubated at 95°C for 10 min, and slowly cooled down to room temperature to anneal the primer to the template. Then, the primer extension reaction was carried out by mixing 50 nM primer/75 nM template, varying concentrations of corresponding faNTP, and 4–10 nM SF WT or SFM5-7 in 1× standard Taq reaction buffer and incubating the mixture at 50°C for 3–20 min. The reactions were quenched by the addition of 2× TBE–urea sample buffer, and the resultant solutions were incubated at 95°C for 10 min. The products were then analyzed with 20% denaturing PAGE gels containing 8 M urea. The bands were quantified with Quantity One software, and the *k*_cat_ and *K*_M_ values were obtained by fitting the data to the Michaelis–Menten equation. All experiments were triplicated.

### Determination of the fidelity of SFM5-7 for FANA synthesis

To determine the fidelity of SFM5-7 for the synthesis of FANA from a DNA template, the synthesis (transcription) of FANA with biotinylated DNA primer B-P18 and DNA template T-RT68 (Supplementary Table S1) by SFM5-7 was carried out as described above. Then, the transcription product was purified with a Zymo ssDNA/RNA Clean & Concentrator™ kit, mixed with SA-coated magnetic beads, and incubated at 37°C overnight. After the incubation, the beads were extensively washed with 100 mM sodium hydroxide (NaOH) to remove the DNA template. Then, the biotinylated chimeric DNA–FANA product was eluted from the beads by resuspending the beads in 98% formamide and incubating the suspension at 95°C for 10 min. The eluted chimeric DNA–FANA product was further purified with a Zymo ssDNA/RNA Clean & Concentrator™ kit, after which the reverse transcription primer Cy3-P20 (Supplementary Table S1) was annealed to the chimeric DNA–FANA product by mixing Cy3-P20 and the chimeric DNA–FANA product in phi29 DNAP reaction buffer, incubating the mixture at 95°C for 10 min, and slowly cooling it down to room temperature. The reverse transcription of the FANA was carried out by mixing 5–20 nM primer Cy3-P20/10–30 nM chimeric DNA–FANA product, 0.5 mM each of dNTPs, and 5 U/μl phi29 DNAP in 1× phi29 DNAP reaction buffer and incubating the mixture at 30°C for 12 h [46]. To test the efficiency of the DNA template removal by NaOH washing, qPCR (quantitative real-time PCR) assay was carried out with the reverse transcription product produced with the protocol described above, and the product produced with the same protocol except that phi29 DNAP was excluded in the reaction solution for reverse transcription. The qPCR mixture was prepared by mixing one of these two products or ddH_2_O, 1 μM each of primers P20-RT and B-P18 (Supplementary Table S1), and 1× SYBR Green qPCR premix, and then subjected to the following thermocycling program: 94°C, 2 min; 30 cycles of (94°C, 30 s; 55°C, 30 s; 72°C, 30 s); and 72°C, 10 min. Next, the DNA product from reverse transcription was PCR amplified with primers EcoRI-F and HindIII-R (Supplementary Table S1) and Q5 DNAP using the following thermocycling program: 94°C, 2 min; 15 cycles of (94°C, 30 s; 55°C, 30 s; 72°C, 30 s); and 72°C, 10 min. The PCR product was purified with a Zymo ssDNA/RNA Clean & Concentrator™ kit, digested with EcoRI and HindIII, and ligated into pET28a vector digested with the same restriction enzymes. The ligation product was transformed into *E*. *coli* DH5α cells, and plasmids were extracted from overnight cultures of the single colonies and sent for sequencing.

### Incorporation of a ribonucleotide into the 3′-end of the self-priming hairpin DNA template

To confirm that under the used reaction conditions, a single ribonucleotide could be incorporated into the 3′-end of the self-priming hairpin DNA template by SFM5-7, FAM-labeled primer FAM-P20 was annealed with template T-rA, T-rU, T-rC, or T-rG (Supplementary Table S1) in standard Taq reaction buffer by incubating the solution at 95°C for 10 min and slowly cooling it down to room temperature. Then, 0.2 μM of the primer/0.3 μM template was mixed with 1 μM of the corresponding rNTP and 1 μM SFM5-7 in 1× standard Taq reaction buffer and incubated at 50°C for 10 min. The reactions were quenched by the addition of 2× TBE–urea sample buffer, and the resultant solutions were incubated at 95°C for 10 min. Then, the products were analyzed with 20% denaturing PAGE gels containing 8 M urea.

The 5′-azide-labeled self-priming hairpin DNA template (T-55U, T-55A, T-55C, or T-55G) (Supplementary Table S1) was folded in standard Taq reaction buffer by incubating the solution at 95°C for 10 min and slowly cooling it down to room temperature. Then, each of these four self-priming hairpin DNA templates was extended with the corresponding rNTP and SFM5-7 to incorporate a ribonucleotide into its 3′ end using the same reaction conditions as described above, and purified with a Zymo ssDNA/RNA Clean & Concentrator™ kit.

### Extension of the self-priming hairpin DNA template harboring the 3′-end ribonucleotide via the synthesis of FANA or another XNA

The purified self-priming hairpin DNA template harboring the 3′-end ribonucleotide was incubated with DBCO-coated magnetic beads at 50°C overnight. After removal of the supernatant, the magnetic beads were thoroughly washed with washing buffer B [1× phosphate buffered saline (PBS), 0.1% Tween 20, pH 7.5] to remove any free DNA template. The magnetic beads, now immobilizing the self-priming hairpin DNA template harboring the 3′-end ribonucleotide, were incubated in standard Taq reaction buffer at 95°C for 2 min and slowly cooled down to room temperature to fold the self-priming hairpin DNA template, and resuspended in the solution for extension reaction, which contained 1 mM each of faNTPs, 5 mM extra MgCl_2_, 1 mM MnCl_2_, and 2.5 μM SFM5-7 in 1× standard Taq reaction buffer. Then, the suspension was incubated at 50°C for 3 h to extend the self-priming hairpin DNA template harboring the 3′-end ribonucleotide via FANA synthesis. For the extension via the synthesis of another XNA, the faNTPs in the reaction solution for extension were replaced with corresponding nucleoside triphosphates, and the reaction time and concentration of MnCl_2_ in the reaction solution were optimized for the best yield and purity of the product. For the synthesis of 2′-F-RNA, the reaction time was shortened to 10 min, and MnCl_2_ was excluded from the reaction solution. For the synthesis of chimeric 2′-F-RNA/2′-OMe-RNA, FANA/DNA, FANA/2′-F-RNA, FANA/2′-F-RNA/2′-OMe-RNA, FANA/DNA/2′-OMe-RNA, or FANA/DNA/2′-F-RNA/2′-OMe-RNA, the reaction time was shortened to 2 h, and MnCl_2_ was excluded from the reaction solution.

### Release and characterization of the FANA or another XNA oligonucleotide product

After the extension of the bead-immobilized self-priming hairpin DNA template harboring the 3′-end ribonucleotide via the synthesis of FANA or another XNA, the supernatant was removed, and the magnetic beads were thoroughly washed with washing buffer B to remove any remaining SFM5-7 and nucleoside triphosphates, resuspended in 500 mM NaOH, and incubated at 65°C for 20 min. Then, the supernatant was collected and neutralized by the addition of hydrochloric acid (HCl), yielding a NaCl solution containing the FANA or another XNA oligonucleotide product. The FANA and other XNA oligonucleotide products were analyzed with 15% denaturing PAGE gels containing 8 M urea. All the gels were stained with Cyber Gold and visualized with a gel imager. The yields of the oligonucleotide products were calculated from the band intensities of the products quantified from the gel images, using a chemically synthesized oligonucleotide with a known concentration as the standard. All the oligonucleotide products were analyzed with high-performance liquid chromatography (HPLC) and liquid chromatography–mass spectrometry (LC–MS).

### Validation of the strategy of regenerating the self-priming hairpin DNA template harboring the 3′-end ribonucleotide with T4 PNK

To validate the strategy of using T4 PNK to regenerate the self-priming hairpin DNA template after NaOH cleavage of the oligonucleotide product, 5′-FAM-labeled DNA primer FAM-P20 was mixed with DNA template T50 in standard Taq reaction buffer, incubated at 95°C for 10 min, and slowly cooled down to room temperature to anneal the primer to the template. Then, the primer extension reaction was carried out by mixing 0.2 μM primer/0.3 μM template, 2 mM rATP, 2 mM rUTP, and 5 μM SFM5-7 in 1× standard Taq reaction buffer, and incubating the mixture at 50°C for 30 min. The extension product was purified with a Zymo ssDNA/RNA Clean & Concentrator™ kit, mixed with 500 mM NaOH, and incubated at 65°C for 20 min. The NaOH cleavage product was neutralized with HCl and purified with a Zymo ssDNA/RNA Clean & Concentrator™ kit. Then, 500 nM NaOH cleavage product was mixed with 5 U/μl T4 PNK or not in 1× T4 PNK buffer and incubated at 37°C for 12 h, and the product was purified with a Zymo ssDNA/RNA Clean & Concentrator™ kit. Then, the primer in the purified product was again annealed to the template by incubating the product in standard Taq reaction buffer at 95°C for 10 min and slowly cooling it down to room temperature, and the annealing product was mixed with 0.5 mM dNTPs and 5 μM SFM5-7 in 1× standard Taq reaction buffer and incubated at 50°C for 30 min to extend the primer. The extension product was again mixed with 500 mM NaOH and incubated at 65°C for 20 min. All the extension and NaOH cleavage products were analyzed with 15% denaturing PAGE gels containing 8 M urea.

To further characterize the self-priming hairpin DNA template from NaOH cleavage before and after regeneration with T4 PNK, self-priming hairpin DNA template B-T-55U (Supplementary Table S1) was extended and incubated with NaOH as described above. Then, the NaOH cleavage product was incubated with T4 PNK or not, purified with a Zymo ssDNA/RNA Clean & Concentrator™ kit, and analyzed with LC–MS.

### Regeneration of the self-priming hairpin DNA template harboring the 3′-end ribonucleotide for recycled production of FANA or another XNA oligonucleotide

After NaOH cleavage to release the FANA or another XNA oligonucleotide product, the magnetic beads were thoroughly washed with washing buffer B to remove remaining NaOH. Then, the magnetic beads were resuspended in 1× T4 PNK buffer containing 5 U/μl T4 PNK and incubated at 37°C for 12 h to remove the 2′ or 3′ phosphate from the 3′-end ribose of the template. After removal of the supernatant, the magnetic beads were thoroughly washed with washing buffer B, resuspended in washing buffer B, and stored at 4°C. For next cycle of FANA or another XNA production, the magnetic beads were directly resuspended in the solution for extension reaction to carry out the template extension via the synthesis of FANA or another XNA. As an example for testing the efficiency of the recycled production of these oligonucleotides, we carried out four cycles of FANA production and analyzed all the products with 15% denaturing PAGE gels containing 8 M urea. The bands were quantified with Quantity One software. All experiments were triplicated.

### Production of 5′-end-labeled FANA oligonucleotides with the SPEXOS platform

To confirm that under the used reaction conditions, a single EdU or dC^Am^ nucleotide or an EdU and a dC^Am^ nucleotides could be incorporated into the 3′ end of the self-priming hairpin DNA template harboring a 3′-end ribonucleotide by SFM5-7, FAM-labeled primer FAM-P20 was annealed with template T-rAEdU, T-rAC^Am^, or T-rA-UC (Supplementary Table S1) and extended with rATP by SFM5-7 to incorporate an rA nucleotide into its 3′ end, and purified with a Zymo ssDNA/RNA Clean & Concentrator™ kit as described above. An EdU or dC^Am^ nucleotide was incorporated into the 3′ end of the primer FAM-P20 harboring the 3′-end rA nucleotide by mixing 0.2 μM of the primer/template (T-rAEdU or T-rAC^Am^) with 1 μM EdUTP or 1 μM dC^Am^TP and 1 μM SFM5-7 in 1× standard Taq buffer and incubating the mixture at 50°C for 2 min. The reactions were quenched by the addition of 2× TBE–urea sample buffer and the resultant solutions were incubated at 95°C for 10 min. An EdU and a dC^Am^ nucleotides were incorporated into the 3′ end of the primer FAM-P20 harboring the 3′-end rA nucleotide by mixing 0.2 μM of the primer/template T-rA-UC with 1 μM EdUTP, 1 μM dC^Am^TP, and 1 μM SFM5-7 in 1× standard Taq buffer and incubating the mixture at 50°C for 2 min. The reactions were quenched by the addition of 2× TBE–urea sample buffer, and the resultant solutions were incubated at 95°C for 10 min. The products were analyzed with 20% denaturing PAGE gels containing 8 M urea.

An rA nucleotide was incorporated into the 3′ end of 5′-azide-labeled self-priming hairpin DNA template T-56-rAU, T-56-rAC, or T-57-rAUC (Supplementary Table S1), respectively, and the products were purified and folded again as described above. To produce a FANA oligonucleotide with a single-labeled 5′ end, an EdU or dC^Am^ nucleotide was incorporated into the 3′ end of template T-56-rAU or T-56-rAC harboring the 3′-end rA nucleotide by mixing 0.2 μM of the template with 1 μM EdUTP or 1 μM dC^Am^TP and 1 μM SFM5-7 in 1× standard Taq buffer and incubating the mixture at 50°C for 2 min. To produce a FANA oligonucleotide with a dual-labeled 5′ end, an EdU and a dC^Am^ nucleotide were incorporated into the 3′ end of template T-57-rAUC harboring the 3′-end rA nucleotide by mixing 0.2 μM of the template with 1 μM EdUTP, 1 μM dC^Am^TP, and 1 μM SFM5-7 in 1× standard Taq buffer and incubating the mixture at 50°C for 2 min. The products with the incorporated ribonucleotide and functionalized deoxyribonucleotide(s) were purified using a Zymo ssDNA/RNA Clean & Concentrator™ kit, immobilized onto the DBCO-coated magnetic beads, and extended via FANA synthesis. Then, the 5′-labeled FANA oligonucleotides were cleaved and washed off with NaOH, neutralized with HCl, and purified as described above. The 5′-EdU-labeled FANA oligonucleotide was mixed with 100 μM CuSO_4_, 500 μM THPTA, 100 μM AF488-azide, and 2.5 mM ascorbic acid, and incubated at 37°C for 30 min. The reaction product was purified with a Zymo ssDNA/RNA Clean & Concentrator™ kit. The 5′-dC^Am^-labeled FANA oligonucleotide was first mixed with 0.1 mM of propargyl-PEG_4_-NHS in PBS (pH 8.5) and incubated at room temperature for 2 h. Then, the reaction product was purified with a Zymo ssDNA/RNA Clean & Concentrator™ kit and further coupled to AF488-azide via click reaction as described above. The FANA oligonucleotide whose 5′ end was labeled with both EdU and dC^Am^ was coupled with AF488-azide and NHS-biotin as described above, and an aliquot of the coupling product was incubated with 2 μg/μl SA at 37°C for 1 h before gel analysis. All the products were then analyzed with 15% denaturing PAGE gels containing 8 M urea, and the gel was directly imaged under blue light without staining.

### Activity verification of the FANAzyme produced with the SPEXOS platform

FANAzyme FR6_1_BRaf600 was produced with the SPEXOS platform as described above, and RNase-free solutions were used throughout the whole process. For the activity assay of FR6_1_BRaf600, 10 μM of FR6_1_BRaf600 was mixed with 2 μM of the RNA substrate Cy3-R30 and 1 mM MgCl_2_ in 10 mM Tris–HCl buffer (pH 7.5) and incubated at 37°C for 15 h to allow the FANAzyme to bind and cleave the RNA substrate. For comparison, the same assay was carried out with FR6_1_BRaf600 synthesized by conventional solid-phase chemical synthesis in parallel. For the control experiment, FR6_1_BRaf600 was excluded in the reaction solution. The products were analyzed with a 15% denaturing PAGE gel containing 8 M urea.

## Results and discussion

### Activities of SF mutants for the synthesis of FANA

**Figure 2.**
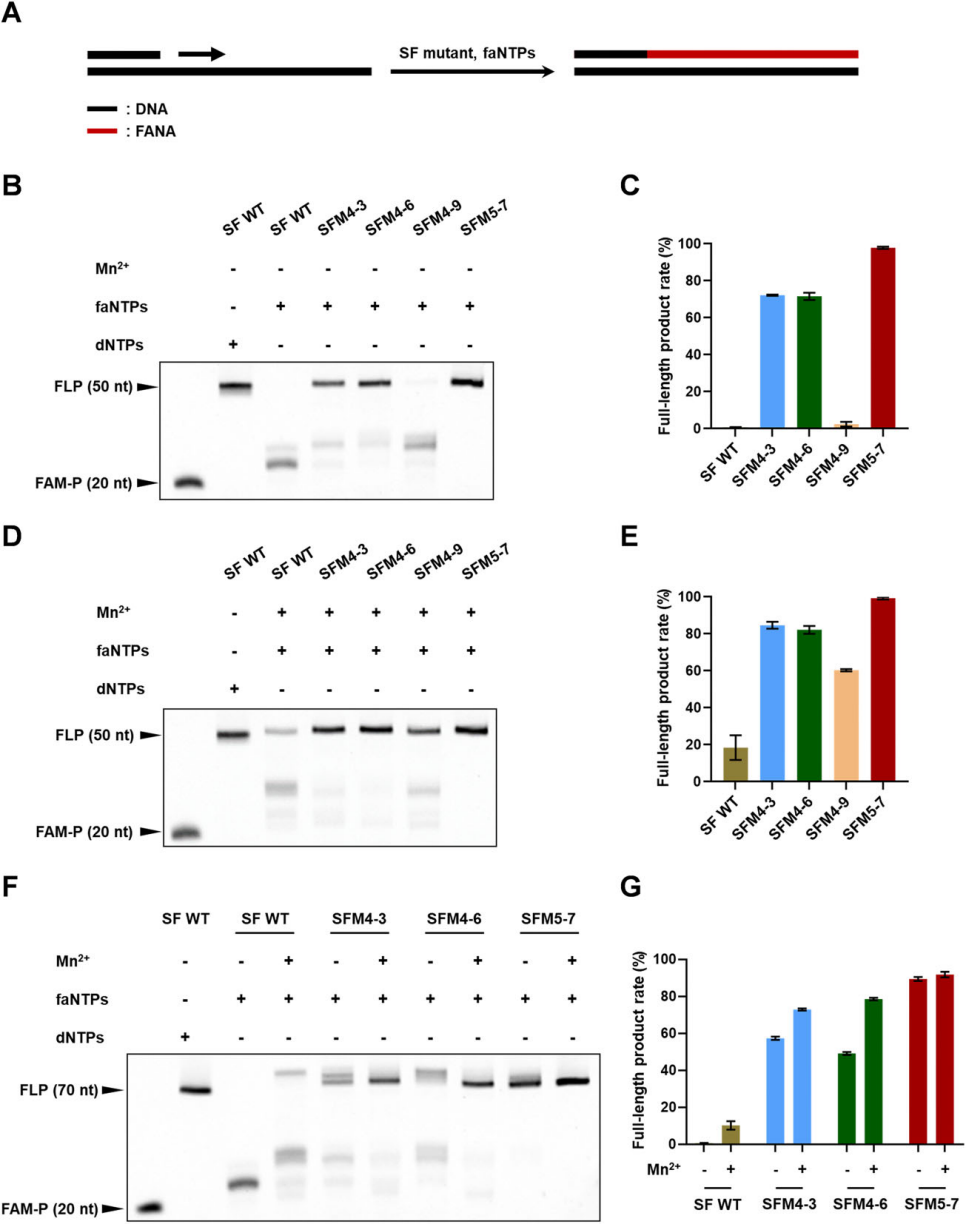
FANA synthesis by different SF mutants. (**A**) Schematic illustration of the FANA synthesis using a DNA template. (**B**) FANA synthesis with a 50-nt DNA template, a 20-nt DNA primer, faNTPs, and SF WT or one of the representative SF mutants in the absence of Mn^2+^. (**C**) Full-length product rates for the FANA synthesis with the 50-nt DNA template, the 20-nt DNA primer, faNTPs, and SF WT or one of the SF mutants in the absence of Mn^2+^. (**D**) FANA synthesis with the 50-nt DNA template, the 20-nt DNA primer, faNTPs, and SF WT or one of the representative SF mutants in the presence of 1 mM Mn^2+^. (**E**) Full-length product rates for the FANA synthesis with the 50-nt DNA template, the 20-nt DNA primer, faNTPs, and SF WT or one of the SF mutants in the presence of 1 mM Mn^2+^. (**F**) FANA synthesis with a 70-nt DNA template, a 20-nt DNA primer, faNTPs, and SF WT or one of the representative SF mutants in the absence or presence of 1 mM Mn^2+^. (**G**) Full-length product rates for the FANA synthesis with the 70-nt DNA template, the 20-nt DNA primer, faNTPs, and SF WT or one of the SF mutants in the presence or absence of 1 mM Mn^2+^. FLP: full-length product for the synthesis of the DNA size marker; FAM-P: FAM-labeled DNA primer FAM-P20 (20 nt) that was used for all of the primer extension experiments.

To establish a SPEXOS platform, we first must identify an efficient XNA polymerase capable of catalyzing accurate XNA synthesis. Currently, known efficient FANA polymerases are predominantly WT or engineered variants of family B DNAPs (Supplementary Table S2), while efficient FANA polymerases derived from family A DNAPs have barely been reported. Previously, SF mutants SFM4-3, SFM4-6, SFM4-9, and SFM5-7 have been evolved to be able to synthesize, reverse transcribe, or amplify 2′-OMe-modified nucleic acids and 2′-F-modified nucleic acids (Supplementary Table S2) [28, 29]. Since these SF mutants demonstrated a significantly expanded substrate spectrum toward nucleotides with varying sugar moieties, we speculated that they might also be active in synthesizing FANA from a DNA template. To test this hypothesis, we expressed and purified the SF WT and the SF mutants using nickel affinity chromatography. Their activities for FANA synthesis were assessed through primer extension experiments with faNTPs (Fig. 1B) and DNA templates of varying lengths (Fig. 2A). After gel analysis of the products, the full-length product rates were calculated from the band intensities of the products quantified from the gel images. First, primer extension experiments were carried out on a 50-nt DNA template (T50) and a 20-nt FAM-labeled DNA primer (FAM-P20), in the absence and presence of 1 mM manganese ion (Mn^2+^). As shown in Fig. 2B–E, all four SF mutants demonstrated higher activity for FANA synthesis compared to SF WT, regardless of the absence or presence of Mn^2+^. In the absence of Mn^2+^, SFM4-3, SFM4-6, and SFM5-7 efficiently produced ∼72.2%, 72.6%, and 97.9% full-length FANA products (+30 nt), respectively, while SF WT and SFM4-9 generated negligible full-length FANA products (Fig. 2B and C). In the presence of 1 mM Mn^2+^, the full-length product rates for SFM4-3 and SFM4-6 rose to ∼84.7% and 82.2%, respectively, while that for SFM5-7 exceeded 99.3% (Fig. 2D and E). Although Mn^2+^ improved synthesis efficiency of SF WT and SFM4-9, their full-length product rates remained lower than those of other three SF mutants.

To investigate the performance of SFM4-3, SFM4-6, and SFM5-7 in synthesizing longer FANA oligonucleotides, primer extension experiments were conducted with a 70-nt DNA template (T70) and primer FAM-P20 in the absence and presence of Mn^2+^. As shown in Fig. 2F and G, SFM4-3 and SFM4-6 produced significantly more truncated products compared to when the shorter template was used. The full-length product rates were 57.3% for SFM4-3 and 49.1% for SFM4-6 in the absence of Mn^2+^, and increased to 72.9% and 78.5%, respectively, when Mn^2+^ was added. Remarkably, SFM5-7 outperformed both mutants, achieving full-length product rates of 89.3% in the absence of Mn^2+^ and 91.8% in the presence of Mn^2+^. Additionally, time course studies revealed that SFM5-7 could produce over 76.2% full-length FANA product within 9 h in the absence of Mn^2+^ and more than 55.4% in just 1 h in the presence of 1 mM Mn^2+^ (Supplementary Fig. S1), indicating its potential for efficient and rapid FANA oligonucleotide synthesis. Overall, SFM5-7 was the most efficient mutant for FANA synthesis under all tested conditions. Based on these results, it was selected for further characterization of its catalytic efficiency and fidelity in FANA synthesis, and for establishing a platform to synthesize FANA and other XNAs.

### Steady-state kinetics of SFM5-7 for incorporating faNTPs

To rigorously characterize the activity of SFM5-7 in FANA synthesis, we carried out steady-state kinetic experiments to quantify the catalytic efficiency of SFM5-7 for incorporating each faNTP opposite the corresponding deoxyribonucleotide in the template. Same experiments were conducted with SF WT for comparison. The Michaelis–Menten plots are shown in Supplementary Figs S2 and S3, and the *k*_cat_, *K*_M_, and *k*_cat_/*K*_M_ values for both SF WT and SFM5-7 are summarized in Table 1. For each faNTP, SFM5-7 exhibited significantly higher catalytic efficiency, with *k*_cat_/*K*_M_ values three to four orders of magnitude greater than those of SF WT, indicating that SFM5-7 synthesizes FANA much more efficiently. The catalytic efficiencies of SFM5-7 for different faNTPs are relatively close and follow the order: faCTP > faGTP > faATP > faUTP.

**Table 1.** Steady-state kinetic constants for the incorporation of each of faNTPs opposite the corresponding deoxyribonucleotide (dX) in the DNA template mediated by SF WT and SFM5-7

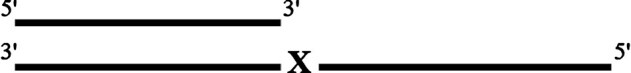					
Polymerase	dX	faNTP	*k*_cat_ (min^−1^)	*K*_M_ (μM)	*k*_cat_/*K*_M_ (M^−1^ min^−1^)
SF WT	dT	faATP	0.060 ± 0.003	61.38 ± 11.59	0.98 × 10^3^
	dA	faUTP	0.030 ± 0.001	18.97 ± 2.63	1.6 × 10^3^
	dG	faCTP	0.19 ± 0.016	423.46 ± 75.32	0.45 × 10^3^
	dC	faGTP	0.38 ± 0.013	242.37 ± 22.87	1.6 × 10^3^
SFM5-7	dT	faATP	0.41 ± 0.005	0.072 ± 0.005	5.7 × 10^6^
	dA	faUTP	0.39 ± 0.013	0.11 ± 0.017	3.5 × 10^6^
	dG	faCTP	0.44 ± 0.009	0.058 ± 0.007	7.6 × 10^6^
	dC	faGTP	0.57 ± 0.012	0.090 ± 0.010	6.3 × 10^6^

### Fidelity of SFM5-7 for FANA synthesis

We further assessed the fidelity of SFM5-7 for FANA synthesis in the absence and presence of Mn^2+^ by measuring the total errors generated during transcription of FANA from a DNA template, reverse transcription of the FANA product back into DNA by phi29 DNAP (Supplementary Fig. S4) [46], and subsequent PCR amplification of the reverse transcription product using Q5 DNAP. FANA transcription was carried out with a biotinylated primer. After transcription, the transcription product was immobilized onto SA-coated magnetic beads, and the DNA template was removed by washing with NaOH. Then, the transcription product was eluted from the beads with pre-heated formamide, purified with a Zymo ssDNA/RNA Clean & Concentrator™ kit, and subjected to reverse transcription. qPCR was performed to monitor the removal efficiency of the original DNA template by NaOH washing and to confirm that the majority of product analyzed originated from reverse transcription of the transcribed FANA. As shown in Supplementary Fig. S5, for both conditions (absence and presence of Mn^2+^), the original DNA template was efficiently removed, with minimal contamination in the reverse transcription product, as evidenced by the significantly higher *C*_q_ value when phi29 DNAP was omitted compared to when both SFM5-7 and phi29 DNAP were included. Subsequently, the reverse transcription product was PCR amplified and cloned into a pET28a vector using EcoRI and HindIII for digestion and T4 ligase for ligation. Following transformation of the ligation product into *E*. *coli* DH5α cells, single colonies were picked for sequencing (Supplementary Fig. S6).

**Figure 3.**
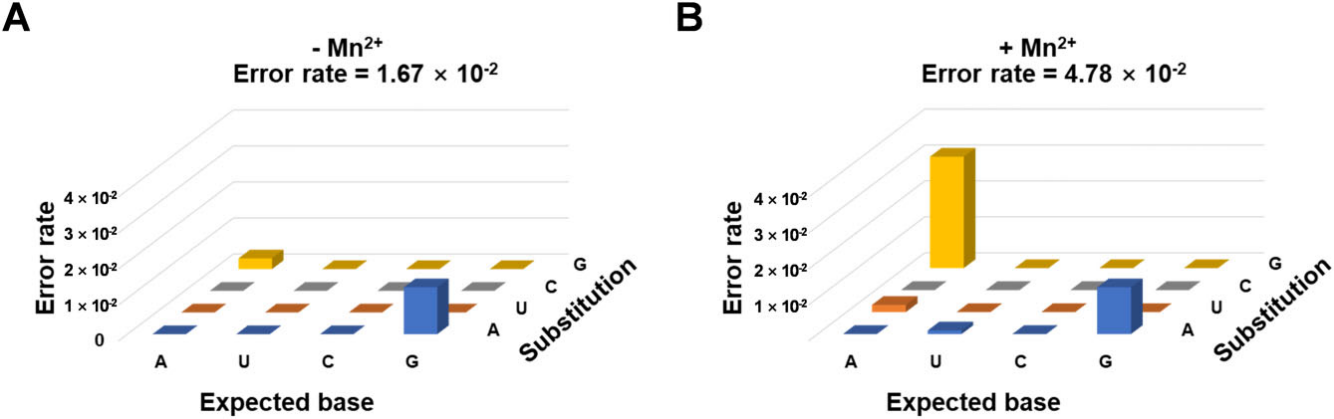
Error profiles for FANA synthesis by SFM5-7. (**A**) Error profile for FANA synthesis by SFM5-7 in the absence of Mn^2+^. (**B**) Error profile for FANA synthesis by SFM5-7 in the presence of 1 mM Mn^2+^.

For each condition (absence or presence of Mn^2+^), a total of 900 bases were sequenced to calculate the overall error rate of FANA transcription, reverse transcription, and PCR amplification. In the absence of Mn^2+^, the overall error rate was found to be 1.67 × 10^–2^, indicating that the error rate for SFM5-7-mediated FANA synthesis is no more than this value. This is comparable to that of FANA synthesis by other engineered FANA polymerases, such as Tgo-D4K [11]. Under this condition, G-to-A mutation was the most frequent (1.33 × 10^–2^), followed by A-to-G mutation (0.33 × 10^–2^) (Fig. 3A). In the presence of Mn^2+^, the overall error rate increased to 4.78 × 10^–2^, with the occurrence rate of G-to-A mutation remained unchanged at 1.33 × 10^–2^, while that of A-to-G mutation rose significantly to 3.11 × 10^–2^, making it the predominant mutation (Fig. 3B). Clearly, SFM5-7 exhibited reasonable fidelity for FANA synthesis, with a notably lower error rate in the absence of Mn^2+^. Although compared to the common fidelity of DNA synthesis by DNA polymerases, the fidelity of FANA synthesis by the polymerase mutants is lower, this level of fidelity for FANA synthesis is adequate for applications that can tolerate or even benefit from minor sequence heterogeneity, such as selection and primary functional test of FANA aptamers and FANAzymes [11, 18–24]. To perfectly satisfy the need for higher fidelity in applications such as production of antisense oligonucleotide drugs, the polymerases can be further engineered to enhance their fidelity while maintaining the synthesis efficiency, which is one of our ongoing efforts in the laboratory. Our work represents the first systematic characterization of the activity and fidelity of an evolved family A DNAP mutant for FANA synthesis. The robust activity and reasonable fidelity of SFM5-7 for FANA synthesis should lay the foundation for its broad applications in evolving FANA aptamers and FANAzymes, as well as in the facile enzymatic preparation of FANA oligonucleotides for synthetic biology, biotechnology, biomedicine, and nanotechnology.

### Establishment of the XNA polymerase-based SPEXOS platform for the streamlined FANA oligonucleotide production

**Figure 4.**
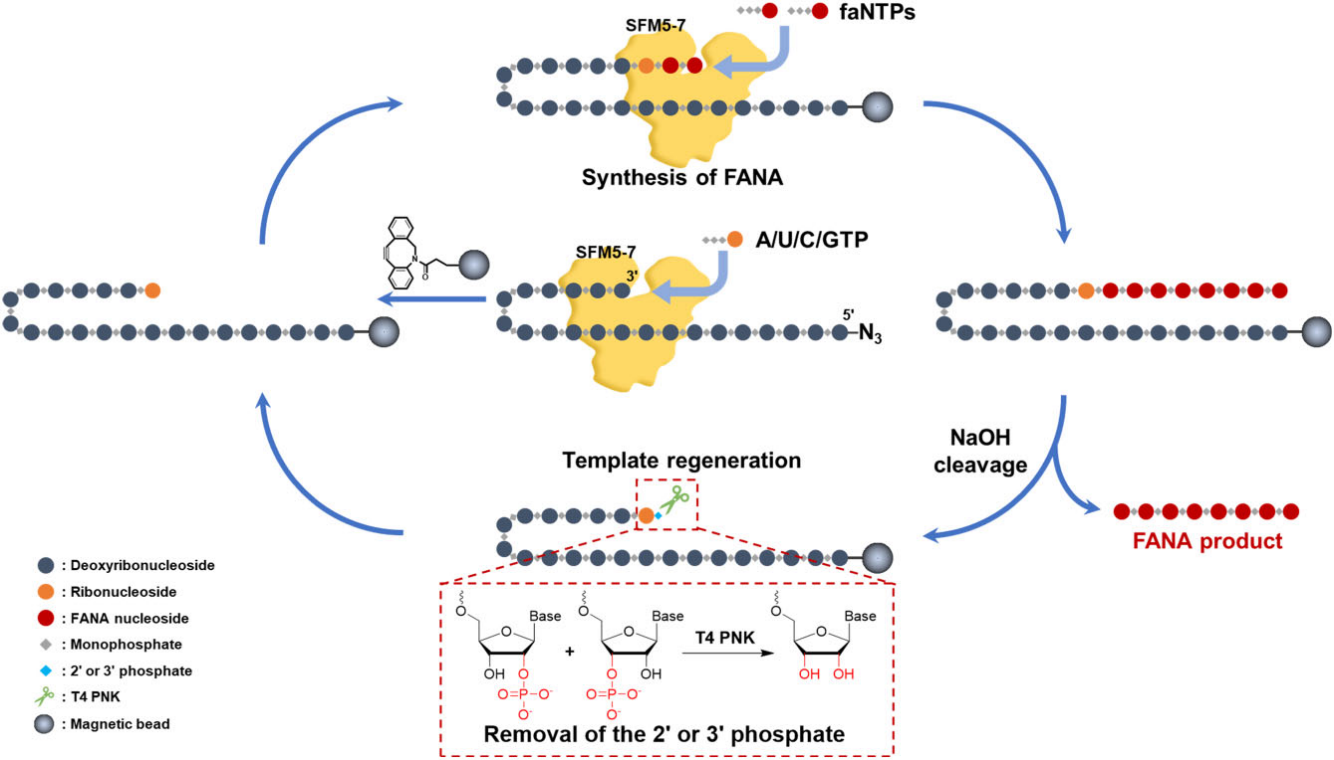
XNA polymerase-based SPEXOS platform for the streamlined production of FANA oligonucleotides.

To broadly utilize FANA in different fields, FANA oligonucleotides have to be easily available. Given that SFM5-7 efficiently synthesizes FANA, DNA, RNA, and several other 2′-modified XNAs [29], we aimed to establish the SPEXOS platform using SFM5-7. This platform is intended to synthesize FANA, other 2′-modified XNAs, and modified or labeled FANA (Fig. 4), potentially addressing the major limitations of existing XNA oligonucleotide synthesis methods. In designing this platform, the 3′ end of a 5′-azide-labeled self-priming hairpin DNA template is first extended with SFM5-7, incorporating a ribonucleotide opposite to the corresponding deoxyribonucleotide in the template. Then, the 5′-end of the template is immobilized onto DBCO-coated magnetic beads via strain-promoted alkyne–azide cycloaddition. Next, the template is further extended with faNTPs by SFM5-7 to synthesize FANA. The beads were incubated and washed with NaOH, which deprotonates the 2′-hydroxy group of the ribonucleotide to cleave its 3′ phosphodiester bond and denatures the FANA/DNA heteroduplex, releasing the cleaved FANA oligonucleotide from the beads (Supplementary Fig. S7). Then, the supernatant containing FANA product is collected and neutralized with HCl, resulting in a NaCl solution suitable for subsequent applications, either directly or after desalting. This approach significantly simplifies purification and reduces loss of the FANA product. Depending on needs, the self-priming hairpin template can be regenerated for further FANA production by removing the 2′ or 3′ phosphate from the ribonucleotide using T4 PNK [25, 47].

To verify the SPEXOS platform design and its versatility for producing FANA oligonucleotides with various sequences, particularly different 5′-end nucleotides, we first assessed SFM5-7’s activity in extending the 3′ end of various self-priming hairpin DNA templates by incorporating a single ribonucleotide opposite the corresponding deoxyribonucleotide using different rNTPs. For this, FAM-labeled primer FAM-P20 was annealed with template T-rA, T-rU, T-rC, or T-rG and extended with rATP, rUTP, rCTP, or rGTP, respectively, by SFM5-7. As shown in Supplementary Fig. S8, SFM5-7 efficiently incorporated a single ribonucleotide into the 3′ end of primer FAM-P20 under the optimized reaction conditions, with no significant by-products detected.

**Figure 5.**
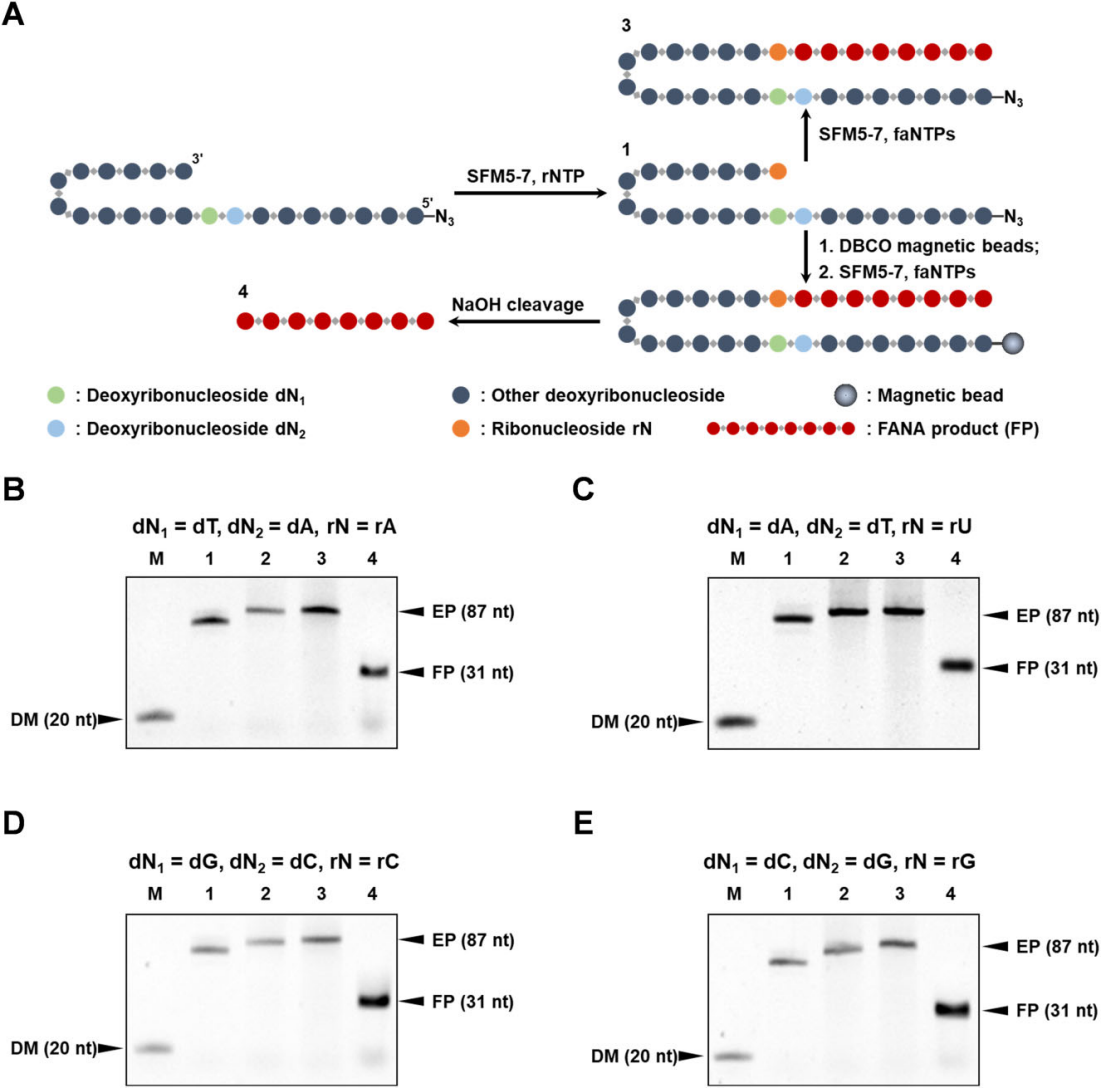
Production of FANA oligonucleotides by the SPEXOS platform. (**A**) Scheme for the production of FANA oligonucleotides with different 5′-end nucleotides. dN_1_ ≠ dN_2_. (**B–E**) Denaturing PAGE analysis of the products of different steps for the production of FANA oligonucleotides with all possible different 5′-end nucleotides using the SPEXOS platform. Lane M: marker; lane 1: the self-priming hairpin DNA template with the ribonucleotide incorporated into the 3′ end; lane 2: the self-priming hairpin DNA template with the ribonucleotide incorporated into the 3′ end and further extended with dNTPs by SFM5-7 (size marker for the full-length extension product); lane 3: the self-priming hairpin DNA template with the ribonucleotide incorporated into the 3′ end and further extended with faNTPs by SFM5-7; lane 4: product cleaved off from the bead-immobilized extension product by NaOH. DM: ssDNA size marker P20 (20 nt); EP: full-length product of extension with faNTPs; FP: FANA oligonucleotide product (31 nt). For each case, the actual nucleotides for dN_1_, dN_2_, and rN illustrated in panel (**A**) are shown above the gel image.

Next, we tested how different incorporated ribonucleotides affect SFM5-7’s efficiency in extending the self-priming hairpin DNA template with faNTPs to synthesize FANA, as well as the efficacy of NaOH cleavage for producing FANA oligonucleotides with different sequences, particularly at the 5′ end. We synthesized four self-priming hairpin DNA templates T-55A, T-55U, T-55C, and T-55G, each containing a dT, dA, dG, or dC opposite where an rA, rU, rC, or rG ribonucleotide would be incorporated, along with a distinct 5′ deoxyribonucleotide (Fig. 5A and Supplementary Fig. S9). Each template was extended with SFM5-7 and the corresponding rNTP, purified with a Zymo ssDNA/RNA Clean & Concentrator™ kit, and either immobilized onto the DBCO-coated magnetic beads or kept non-immobilized for gel analysis. Subsequently, the template incorporating the ribonucleotide was further extended with SFM5-7 and faNTPs to synthesize the FANA oligonucleotide. For gel analysis, we prepared a size marker by further extending the non-immobilized template using dNTPs. The FANA product was cleaved from the self-priming hairpin template by washing the beads with 0.5 M NaOH, and then the supernatant containing the FANA product was neutralized with HCl. The resulting products were analyzed using denaturing PAGE and stained with Cyber Gold. As shown in Fig. 5B–E, all four templates were efficiently extended to full length with faNTPs by SFM5-7, producing FANA oligonucleotides with distinct 5′-end nucleotides at high purity upon NaOH cleavage of the extension product, evident from the sharp and clear product bands on the gels. As an example, the FANA oligonucleotide produced from self-priming hairpin DNA templates T-55U was selected for further yield calculation and characterization. Based on the gel analysis of the product amount, from 100.0 pmol template, 56.3 pmol FANA was produced, suggesting a yield of 56.3% was achieved. We also characterized this FANA oligonucleotide product with HPLC and LC–MS. As shown in Supplementary Fig. S10, the result of the HPLC analysis suggested that the purity of this FANA oligonucleotide product is higher than 95% and the result of the LC–MS analysis further confirmed its identity and integrity. Overall, the incorporation of ribonucleotides, FANA synthesis, and NaOH cleavage all proceeded efficiently, confirming the successful establishment and versatility of the SPEXOS platform for producing FANA oligonucleotides with diverse sequences.

### Production of other XNA and chimeric XNA oligonucleotides with the SPEXOS platform

**Figure 6.**
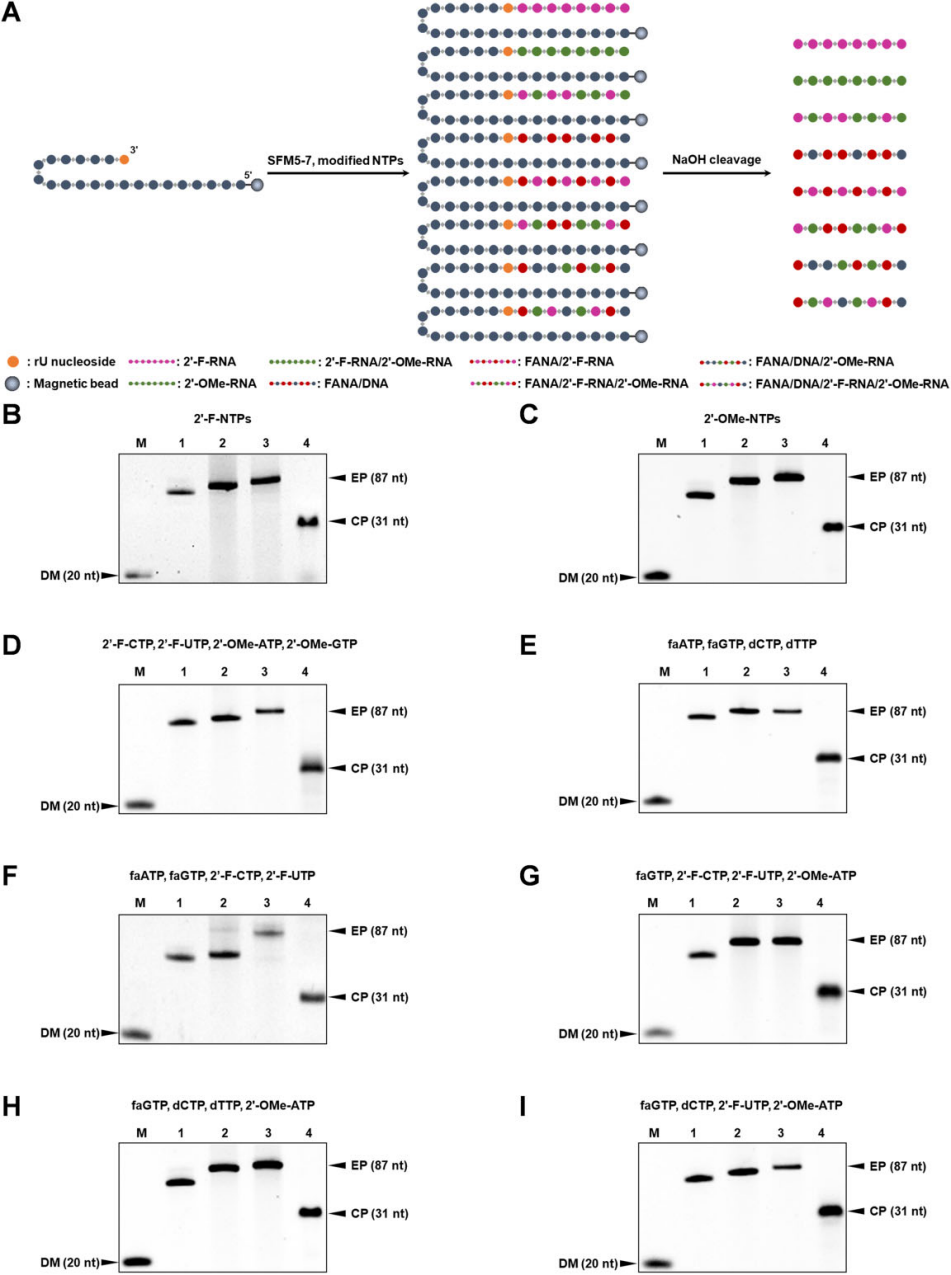
Production of other XNA and chimeric XNA oligonucleotides by the SPEXOS platform. (**A**) Scheme for the production of 2′-F-RNA, 2′-OMe-RNA, chimeric 2′-F-RNA/2′-OMe-RNA, FANA/DNA, FANA/2′-F-RNA, FANA/2′-F-RNA/2′-OMe-RNA, FANA/DNA/2′-OMe-RNA, and FANA/DNA/2′-F-RNA/2′-OMe-RNA oligonucleotides by the SPEXOS platform. (**B–E**) Denaturing PAGE analysis of the products of different steps for the production of other XNA and chimeric XNA oligonucleotides with the SPEXOS platform, where the self-priming hairpin DNA template with an rU nucleotide incorporated into the 3′ end (T-55U, Supplementary Table S1) was used. Lane M: marker; lane 1: the self-priming hairpin DNA template with an rU nucleotide incorporated into the 3′ end; lane 2: the self-priming hairpin DNA template with an rU nucleotide incorporated into the 3′ end and further extended with dNTPs by SFM5-7 (size marker for the full-length extension product); lane 3: the self-priming hairpin DNA template with an rU nucleotide incorporated into the 3′ end and further extended with 2′-F-NTPs (**B**), 2′-OMe-NTPs (**C**), a combination of 2′-F-CTP, 2′-F-UTP, 2′-OMe-ATP, and 2′-OMe-GTP (**D**), a combination of faATP, faGTP, dCTP, and dTTP (**E**), a combination of faATP, faGTP, 2′-F-CTP, and 2′-F-UTP (**F**), a combination of faGTP, 2′-F-CTP, 2′-F-UTP, and 2′-OMe-ATP (**G**), a combination of faGTP, dCTP, dTTP, and 2′-OMe-ATP (**H**), or a combination of faGTP, dCTP, 2′-F-UTP, and 2′-OMe-ATP (**I**) by SFM5-7; lane 4: product cleaved off from the bead-immobilized extension product by NaOH. DM: ssDNA size marker P20 (20 nt); EP: full-length product of extension with a combination of sugar-modified nucleoside triphosphates (87 nt); CP: XNA or chimeric XNA oligonucleotide product produced by NaOH cleavage (31 nt).

Based on the great activity of SFM5-7 in synthesizing DNA, FANA, and other 2′-modified XNAs, we anticipated that the SPEXOS platform could also be adapted for producing other 2′-modified XNA oligonucleotides, as well as chimeric XNAs formed by combining different XNA nucleotides. To test this, we produced 2′-F-RNA and 2′-OMe-RNA, along with chimeric 2′-F-RNA/2′-OMe-RNA, FANA/DNA, FANA/2′-F-RNA, FANA/2′-F-RNA/2′-OMe-RNA, FANA/DNA/2′-OMe-RNA, and FANA/DNA/2′-F-RNA/2′-OMe-RNA oligonucleotides by substituting faNTPs with various combinations of nucleoside triphosphates (Fig. 1B) during the extension of the self-priming hairpin DNA template T-55U incorporating a 3′-end rU nucleotide (Fig. 6A). As shown in Fig. 6B and C, and Supplementary Figs S11 and S12, SFM5-7 efficiently extended the template with 2′-F-NTPs or 2′-OMe-NTPs, producing 2′-F-RNA and 2′-OMe-RNA oligonucleotides with purities exceeding 95% and yields of 68.7% and 46.8%, respectively. The result of the LC–MS analysis further confirmed the identity and integrity of these oligonucleotide products, and revealed that for the 2′-F-RNA oligonucleotide product, a non-templated 2′-F-A nucleotide was incorporated into its 3′ end. This indicates the SPEXOS platform’s universality for producing various 2′-modified XNAs beyond FANA. Chimeric oligonucleotides composed of different nucleotides have broad applications in biotechnology and biomedicine [32–34, 48–51], and their successful production would enhance the value of the SPEXOS platform. As shown in Fig. 6D–I and Supplementary Figs S13–S18, extending the template with combinations of different nucleoside triphosphates also led to the efficient production of chimeric 2′-F-RNA/2′-OMe-RNA, FANA/DNA, FANA/2′-F-RNA, FANA/2′-F-RNA/2′-OMe-RNA, FANA/DNA/2′-OMe-RNA, and FANA/DNA/2′-F-RNA/2′-OMe-RNA oligonucleotides with purities exceeding 95% and yields of 61.0%, 63.6%, 66.3%, 58.0% 53.9%, and 59.2%, respectively. The result of the LC–MS analysis again further confirmed the identity and integrity of these oligonucleotide products (Supplementary Figs S13–S18). It is worth mentioning that most of these oligonucleotides cannot be produced using strategies that involve DNase I to remove the DNA primer and template, as they contain nucleotide components vulnerable to DNase I degradation, i.e. deoxyribonucleotides and FANA nucleotides. Despite the value of the SPEXOS platform in producing chimeric oligonucleotides, it should be noted that only chimeric oligonucleotides in which all the nucleotides of the same base share the same sugar modification can be produced with this platform. While this precludes the production of therapeutic oligonucleotides in which some nucleotides of the same base possess different sugar modifications, it is ideally suited for generating chimeric XNA libraries for selection, or chimeric XNA aptamers and XNAzymes with enhanced biostability and functional diversity. Another limitation of this platform is that chimeric oligonucleotides containing ribonucleotides are not suitable to be synthesized with this platform, due to the employment of NaOH for product cleavage and release. Overall, the SPEXOS platform provides an efficient and facile method for synthesizing not only FANA and other XNA oligonucleotides, but also chimeric XNA oligonucleotides with various nucleotide combinations with good purity and yield.

### Recycled XNA oligonucleotide synthesis with the SPEXOS platform

In the SPEXOS platform, NaOH cleavage of the 3′ phosphodiester bond of the ribonucleotide minimally damages the bead-immobilized self-priming hairpin DNA template, allowing it to be reused in subsequent cycles of XNA oligonucleotide production. To regenerate the template, the 2′ or 3′ phosphate on the 3′-end ribonucleotide generated during NaOH cleavage should be removed (Fig. 4 and Supplementary Fig. S7) [25]. T4 PNK has been shown to efficiently eliminate these phosphates, enabling the template’s reuse [47]. To verify this, we annealed FAM-labeled primer FAM-P20 with template T50 and extended it with rATP and rUTP using SFM5-7 (Supplementary Fig. S19A). The extension product was then cleaved with NaOH, resulting in a phosphorylated rU nucleotide at the 3′ end of the primer. The NaOH cleavage product was neutralized and purified, and then treated with T4 PNK or left untreated. The primer was subsequently annealed to the template again and tested for extension with dNTPs. As shown in Supplementary Fig. S19B, the untreated primer could not be extended by SFM5-7, while that treated with T4 PNK could be efficiently extended to produce the full-length product. Subsequent NaOH cleavage of the re-extension product effectively released the oligonucleotide product, evidenced by a sharp band on the gel.

To confirm the change in the 3′-end ribonucleotide of the NaOH-cleaved self-priming hairpin DNA template after T4 PNK treatment, we extended self-priming hairpin DNA template B-T-55U with rATP, rUTP, and SFM5-7, cleaved the extension product with NaOH, and analyzed the cleavage product treated with T4 PNK or not by LC–MS (Supplementary Fig. S20). The mass spectra revealed that the untreated product retained its phosphate group, while the T4 PNK-treated product displayed hydroxyl (OH) groups at the 2′ and 3′ positions, confirming the phosphate removal by T4 PNK. These findings support the feasibility and efficiency of regenerating the self-priming hairpin DNA template by T4 PNK treatment after NaOH cleavage.

**Figure 7.**
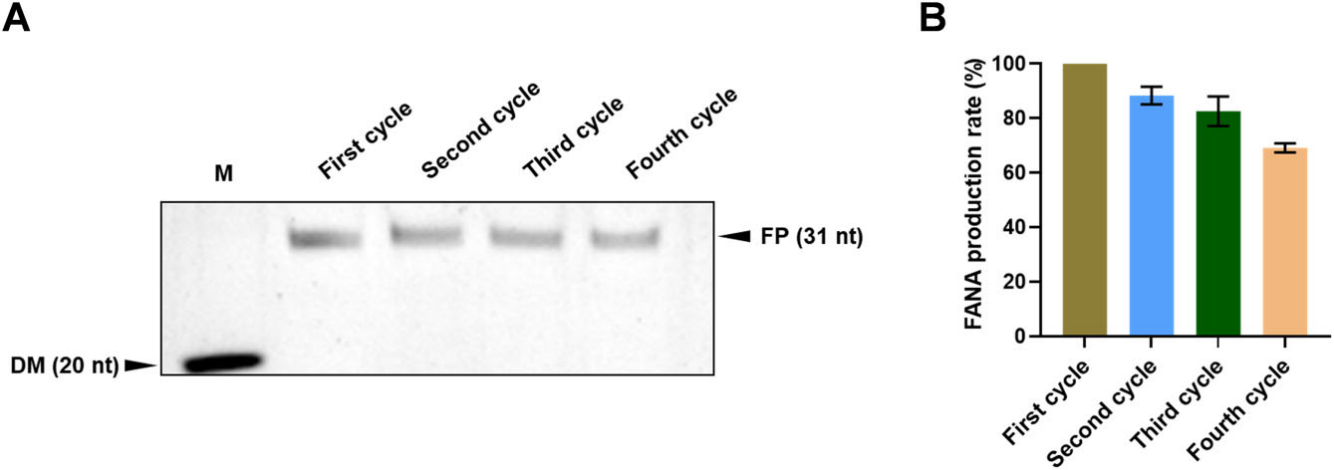
Recycled production of a FANA oligonucleotide with the SPEXOS platform. The FANA oligonucleotide was produced for four cycles by reusing the bead-immobilized self-priming hairpin DNA template. (**A**) Denaturing PAGE analysis of the FANA oligonucleotide produced in different cycles of production with the SPEXOS platform. Lane M: marker. DM: ssDNA size marker P20 (20 nt); FP: FANA oligonucleotide product (31 nt). (**B**) Production rates of the FANA oligonucleotide in different cycles of production. The production rate in the first cycle was defined as 100%.

Based on this, the SPEXOS platform could be employed for recycled XNA oligonucleotide production, using a bead-immobilized template across multiple cycles. For instance, four cycles of FANA oligonucleotide production efficiently yielded target products without significant by-products in each cycle, with product yields of 88.4%, 82.6%, and 69.2% for the second, third, and fourth cycles compared to the first cycle (Fig. 7A and B). Although as a proof of concept, only 12.9–18.6 pmol oligonucleotide was produced in each cycle of the production, less than that demonstrated in the strategy recently reported by Lovelock and coworkers [38], the oligonucleotide production by the SPEXOS platform should have good scalability, due to the low cost for product cleavage and release by NaOH, as well as replaceability of the magnetic beads with other solid-phase carriers suitable for large-scale production. Additionally, different combinations of nucleoside triphosphates can be used in subsequent cycles, enabling the convenient production of oligonucleotides with the same sequence but distinct modifications. This versatility facilitates the exploration of how these modifications affect the properties and functions of oligonucleotides for various biological applications.

### Production of 5′-end-labeled FANA oligonucleotides with the SPEXOS platform

Labeling of XNA oligonucleotides with functional groups, such as fluorophores, reactive groups, and affinity ligands, is crucial for the identification and characterization of functional XNA molecules. This labeling is essential for various applications, including the preparation of XNA probes, construction of XNA-drug conjugates, and development of XNA sensors and devices [52–59]. Previously, we have demonstrated efficient 3′-labeling of ssDNA and ssXNA with (d)NTPs with functionalized nucleobase via terminal deoxynucleotidyl transferase (TdT) [56, 59]. However, techniques for 5′-end labeling of XNA oligonucleotides remain largely unexplored.

**Figure 8.**
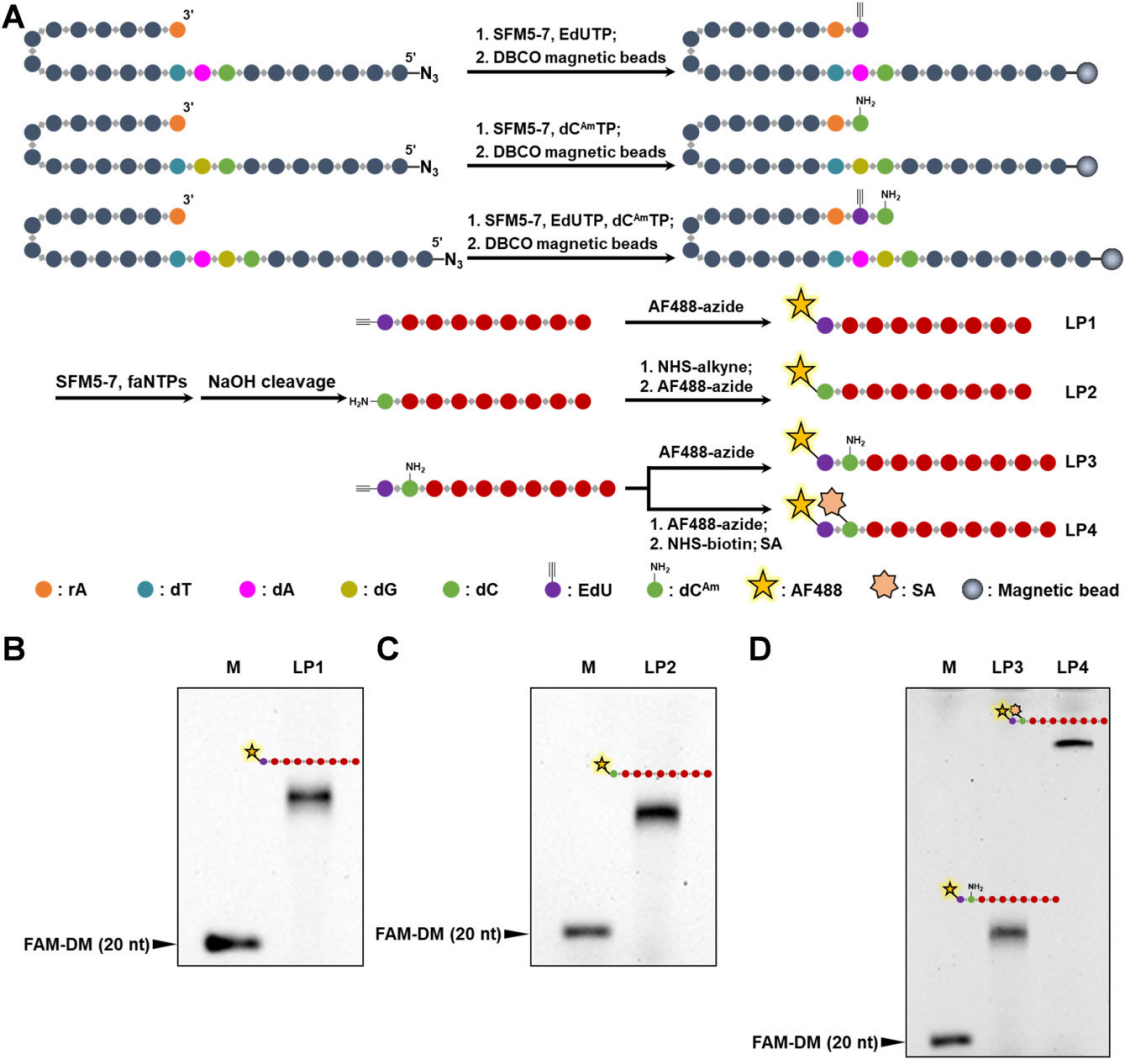
Production of FANA oligonucleotides with single- or dual-labeled 5′ end using the SPEXOS platform. (**A**) Scheme for the production of FANA oligonucleotides 5′-end-labeled with one or two functional groups using the SPEXOS platform. (**B**) Denaturing PAGE analysis of the FANA oligonucleotide product 5′-end-labeled with EdU and subsequently coupled with AF488-azide (lane LP1). (**C**) Denaturing PAGE analysis of the FANA oligonucleotide product 5′-end-labeled with dC^Am^ and subsequently coupled with NHS-alkyne and AF488-azide, successively (lane LP2). (**D**) Denaturing PAGE analysis of the FANA oligonucleotide products 5′-end-labeled with both EdU and dC^Am^, and subsequently coupled with only AF488-azide (lane LP3), or coupled with both AF488-azide and NHS-biotin and bound with SA (lane LP4). Lane M in each gel: marker. FAM-DM: FAM-labeled ssDNA size marker FAM-P20 (20 nt).

Here, we explored the application of the SPEXOS platform for the enzymatic production of 5′-end-labeled XNA oligonucleotides, specifically 5′-end-labeled FANA oligonucleotides (Fig. 8A). For this, a self-priming hairpin DNA template was designed to have different nucleotides at positions opposite where the first and second nucleotides will be incorporated, and different nucleotides at positions opposite where the second and third nucleotides will be incorporated when the primer is extended. For the dual labeling experiment, the nucleotides at positions opposite where the third and fourth nucleotides will be incorporated are also different. We first investigated the activity of SFM5-7 for extending a DNA primer with an rA nucleotide at the 3′ end by incorporating an EdU or dC^Am^ nucleotide, or both, opposite the corresponding deoxyribonucleotide(s) in the template with corresponding base-modified dNTPs. This step is crucial for producing XNA oligonucleotides 5′-end-labeled with EdU, dC^Am^, or both (Supplementary Fig. S21). FAM-labeled primer FAM-P20 was annealed with template T-rAEdU, T-rAC^Am^, or T-rA-UC and extended with rATP by SFM5-7 to incorporate an rA nucleotide at the 3′ end. The product was purified with a Zymo ssDNA/RNA Clean & Concentrator™ kit. Then, the extended primer was further extended, respectively, with EdUTP (for template T-rAEdU), dC^Am^TP (Fig. 1B) (for template T-rAC^Am^), or both (for template T-rA-UC) by SFM5-7. The products were analyzed with denaturing PAGE gels. As shown in Supplementary Fig. S21, under the optimized concentrations, SFM5-7 efficiently extended primer FAM-P20 with an rA nucleotide at the 3′ end, accurately incorporating a single EdU (for T-rAEdU) or dC^Am^ (for T-rAC^Am^) nucleotide, or both (for T-rA-UC) with no undesired by-products observed.

Since EdUTP and dC^Am^TP can be efficiently incorporated by SFM5-7, we investigated the SPEXOS platform for the enzymatic production of XNA oligonucleotides with single or dual 5′-end labels using these two nucleoside triphosphates. An rA nucleotide was incorporated into the 3′ end of self-priming hairpin DNA templates T-56-rAU, T-56-rAC, and T-57-rAUC (Supplementary Fig. S22), followed by extension with EdUTP for T-56-rAU, dC^Am^TP for T-56-rAC, or both for T-57-rAUC, to incorporate base-modified deoxyribonucleotides into the 3′-end of the template (Fig. 8A and Supplementary Fig. S22). After immobilized on the beads, the templates were further extended with faNTPs by SFM5-7 for FANA synthesis, and the products were cleaved with NaOH to release the labeled FANA oligonucleotides.

To assess the production efficiency and demonstrate further functionalization of the oligonucleotide products, the 5′-EdU-labeled FANA oligonucleotide was coupled to AF488-azide via a click reaction, while the 5′-dC^Am^-labeled FANA was first linked to an alkyne-NHS linker and then to AF488-azide. The products were analyzed using denaturing PAGE, showing bright, sharp bands that indicate high purity and effective coupling with the fluorophore (Fig. 8B and C). For dual labeling, the product labeled with both EdU and dC^Am^ was coupled with AF488-azide, or both AF488-azide and NHS-biotin with subsequent binding to SA before gel analysis. The resulting bright bands at expected positions suggest the successful incorporation of the EdU and dC^Am^ nucleotides and their efficient coupling. In summary, the SPEXOS platform enables the efficient production of FANA oligonucleotides labeled at the 5′ end with either single or dual functional groups, broadening its application scope for producing 5′-labeled XNA oligonucleotides.

### Production of a functional FANAzyme with the SPEXOS platform

XNA oligonucleotides with specific sequences can serve as functional molecules, such as antisense oligonucleotides, aptamers, and ribozymes, offering improved properties and expanded functions in biotechnology and biomedicine [10, 17–23]. To explore the SPEXOS platform’s potential for the facile production of functional XNA oligonucleotides, we produced an RNA endonuclease FANAzyme, FR6_1_BRaf600, which is known for its excellent biostability, catalytic efficiency, and substrate specificity (Supplementary Fig. S23A and B) [10]. The FANAzyme (10 μM) produced by the SPEXOS platform was directly mixed with 2 μM of the Cy3-labeled RNA substrate and 1 mM MgCl_2_ in 10 mM Tris–HCl (pH 7.5), and incubated at 37°C for 15 h. For comparison, the same experiment was carried out with FR6_1_BRaf600 synthesized by conventional solid-phase chemical synthesis. Analysis of the products via denaturing PAGE revealed that under the same conditions, the RNA cleavage efficiency of the FANAzyme produced by the SPEXOS platform was nearly identical to that of the FANAzyme produced by solid-phase chemical synthesis, which was consistent with that reported in the literature (Supplementary Fig. S23C) [12], suggesting that the FANAzyme prepared by the SPEXOS platform well retained its structural integrity and activity. This clearly demonstrates that functional XNA molecules can be conveniently prepared via the SPEXOS platform. By using different nucleoside triphosphates in various production cycles, it is possible to produce functional XNA molecules with the same sequence but varying modifications with the SPEXOS platform, allowing for investigations into the effect of these modifications on the functionality of XNA molecules.

## Conclusions

In this work, we developed a facile SPEXOS platform using a laboratory-evolved XNA polymerase. For this, we first explored the activity of a series of artificially evolved mutants of the Stoffel fragment of a representative family A DNAP, Taq DNAP, for the synthesis of FANA with different lengths, and identified mutant SFM5-7 as the most efficient. The FANA synthesis activity of SFM5-7 was systematically characterized, revealing its superior ability to incorporate faNTPs compared to the wild-type SF. Additionally, sequencing of the FANA transcription–reverse transcription–PCR amplification product demonstrated SFM5-7’s reasonable fidelity for FANA synthesis. Based on the profound characterization of SFM5-7 and its excellent efficiency in synthesizing DNA, RNA, FANA, and other 2′-modified XNAs, we next developed the SPEXOS platform for the efficient and facile production of XNA oligonucleotides, particularly FANA oligonucleotides. This platform utilizes ribonucleotide incorporation and alkaline cleavage of the 3′ phosphodiester bond of the ribonucleotide to facilitate the easy release of newly synthesized XNA from the self-priming hairpin DNA template. It provides a simple and sustainable means for the production of XNA oligonucleotides, with good product yield and purity, wide product scope, low production cost, and minimized organic waste. Leveraging the expanded substrate repertoire of SFM5-7, this platform’s versatility was validated by the successful production of various XNA oligonucleotides, including FANA, 2′-F-RNA, 2′-OMe-RNA, and chimeric XNAs such as 2′-F-RNA/2′-OMe-RNA, FANA/DNA, FANA/2′-F-RNA, FANA/2′-F-RNA/2′-OMe-RNA, FANA/DNA/2′-OMe-RNA, and FANA/DNA/2′-F-RNA/2′-OMe-RNA. The SPEXOS platform also demonstrated its capability to produce 5′-end-labeled XNA oligonucleotides efficiently, enabling the production and functionalization of FANA oligonucleotides labeled with EdU, dC^Am^, or both. This capability provides a valuable tool for convenient labeling and functionalization of XNA oligonucleotides, facilitating broader applications of XNA in biosensing, bioimaging, bioengineering, and biomedicine. Finally, the SPEXOS platform successfully produced an active FANAzyme, highlighting its potential for the convenient production of functional XNA molecules for immediate evaluation and application. Although relatively small-scale productions of oligonucleotides were demonstrated in this work, the oligonucleotide production by the SPEXOS platform should have good scalability, allowing its broad use in various application scenarios. Despite all the advantages, it should be noted that the SPEXOS platform also has several limitations that should be taken into consideration in its application. First, when this platform is used for producing chimeric oligonucleotides, only oligonucleotides in which the nucleotides of the same base possess the same sugar modification can be produced. Second, oligonucleotides containing ribonucleotides cannot be produced with this platform. Third, for oligonucleotide synthesis that needs extraordinary sequence accuracy, the employed XNA polymerase has to be further engineered to have higher fidelity for XNA synthesis while retaining good synthesis efficiency. In summary, the XNA synthesis platform established in this work enhances the customized production and labeling of XNA oligonucleotides with programmable modifications, promoting the development of XNA-based diagnostics, therapeutics, synthetic biology, and nanotechnology.

## Supplementary Material

gkaf567_Supplemental_File

## Data Availability

The data underlying this article are available in the article and in its online supplementary data.

## References

[1] Duffy K, Arangundy-Franklin S, Holliger P Modified nucleic acids: replication, evolution, and next-generation therapeutics. BMC Biol. 2020; 18:112 10.1186/s12915-020-00803-6. 32878624 PMC7469316

[2] Sun L, Ma X, Zhang B et al. From polymerase engineering to semi-synthetic life: artificial expansion of the central dogma. RSC Chem Biol. 2022; 3:1173–97. 10.1039/D2CB00116K. 36320892 PMC9533422

[3] Chaput JC, Herdewijn P What is XNA. Angew Chem Int Ed. 2019; 58:11570–2. 10.1002/anie.201905999. 31210402

[4] Zhu G, Song P, Wu J et al. Application of nucleic acid frameworks in the construction of nanostructures and cascade biocatalysts: recent progress and perspective. Front Bioeng Biotechnol. 2022; 9:792489 10.3389/fbioe.2021.792489. 35071205 PMC8777461

[5] Majlessi M, Nelson NC, Becker MM Advantages of 2′-*O*-methyl oligoribonucleotide probes for detecting RNA targets. Nucleic Acids Res. 1998; 26:2224–9. 10.1093/nar/26.9.2224. 9547284 PMC147516

[6] Tu T, Huan S, Ke G et al. Functional xeno nucleic acids for biomedical application. Chem Res Chin Univ. 2022; 38:912–8. 10.1007/s40242-021-2186-7. PMC9253239 35814030

[7] Pinheiro VB, Holliger P Towards XNA nanotechnology: new materials from synthetic genetic polymers. Trends Biotechnol. 2014; 32:321–8. 10.1016/j.tibtech.2014.03.010. 24745974 PMC4039137

[8] Damha MJ, Wilds C, Noronha A et al. Hybrids of RNA and arabinonucleic acids (ANA and 2′F-ANA) are substrates of ribonuclease H. J Am Chem Soc. 1998; 120:12976–7. 10.1021/ja982325.

[9] Wright JA, Taylor NF, Fox JJ Nucleosides. LX. Fluorocarbohydrates. XXII. Synthesis of 2-deoxy-2-fluoro-D-arabinose and 9-(2-deoxy-2-fluoro-α- and -β-D-arabinofuranosyl)adenines. J Org Chem. 1969; 34:2632–6. 10.1021/jo01261a031. 5803811

[10] Peng CG, Damha MJ Polymerase-directed synthesis of 2′-deoxy-2′-fluoro-β-D-arabinonucleic acids. J Am Chem Soc. 2007; 129:5310–1. 10.1021/ja069100g. 17419631

[11] Pinheiro VB, Taylor AI, Cozens C et al. Synthetic genetic polymers capable of heredity and evolution. Science. 2012; 336:341–4. 10.1126/science.1217622. 22517858 PMC3362463

[12] Taylor AI, Wan CJK, Donde MJ et al. A modular XNAzyme cleaves long, structured RNAs under physiological conditions and enables allele-specific gene silencing. Nat Chem. 2022; 14:1295–305. 10.1038/s41557-022-01021-z. 36064973 PMC7613789

[13] Taylor AI, Beuron F, Peak-Chew S-Y et al. Nanostructures from synthetic genetic polymers. ChemBioChem. 2016; 17:1107–10. 10.1002/cbic.201600136. 26992063 PMC4973672

[14] Wang Q, Chen X, Li X et al. 2′-Fluoroarabinonucleic acid nanostructures as stable carriers for cellular delivery in the strongly acidic environment. ACS Appl Mater Interfaces. 2020; 12:53592–7. 10.1021/acsami.0c11684. 33206496

[15] Dowler T, Bergeron D, Tedeschi A-L et al. Improvements in siRNA properties mediated by 2′-deoxy-2′-fluoro-β-D-arabinonucleic acid (FANA). Nucleic Acids Res. 2006; 34:1669–75. 10.1093/nar/gkl033. 16554553 PMC1409815

[16] Shukla S, Sumaria CS, Pradeepkumar PI Exploring chemical modifications for siRNA therapeutics: a structural and functional outlook. ChemMedChem. 2010; 5:328–49. 10.1002/cmdc.200900444. 20043313

[17] Damha MJ, Noronha A, Wilds C et al. Properties of arabinonucleic acids (ANA & 20′F-ANA): implications for the design of antisense therapeutics that invoke RNase H cleavage of RNA. Nucleosides Nucleotides Nucleic Acids. 2001; 20:429–40. 11563058 10.1081/NCN-100002317

[18] Alves Ferreira-Bravo I, DeStefano JJ Xeno-nucleic acid (XNA) 2′-fluoro-arabino nucleic acid (FANA) aptamers to the receptor-binding domain of SARS-CoV-2 S protein block ACE2 binding. Viruses. 2021; 13:1983. 34696413 10.3390/v13101983 PMC8539646

[19] Alves Ferreira-Bravo I, Cozens C, Holliger P et al. Selection of 2′-deoxy-2′-fluoroarabinonucleotide (FANA) aptamers that bind HIV-1 reverse transcriptase with picomolar affinity. Nucleic Acids Res. 2015; 43:9587–99. 26476448 10.1093/nar/gkv1057 PMC4751925

[20] Rose KM, Alves Ferreira-Bravo I, Li M et al. Selection of 2′-deoxy-2′-fluoroarabino nucleic acid (FANA) aptamers that bind HIV-1 integrase with picomolar affinity. ACS Chem Biol. 2019; 14:2166–75. 31560515 10.1021/acschembio.9b00237 PMC7005942

[21] Razi N, Li W, Ignacio MA et al. Inhibition of SARS-CoV-2 infection in human airway epithelium with a xeno-nucleic acid aptamer. Respir Res. 2023; 24:272 10.1186/s12931-023-02590-4. 37932762 PMC10629106

[22] Taylor AI, Pinheiro VB, Smola MJ et al. Catalysts from synthetic genetic polymers. Nature. 2015; 518:427–30. 10.1038/nature13982. 25470036 PMC4336857

[23] Wang Y, Ngor AK, Nikoomanzar A et al. Evolution of a general RNA-cleaving FANA enzyme. Nat Commun. 2018; 9:5067 10.1038/s41467-018-07611-1. 30498223 PMC6265334

[24] Wang Y, Vorperian A, Shehabat M et al. Evaluating the catalytic potential of a general RNA-cleaving FANA enzyme. ChemBioChem. 2020; 21:1001–6. 10.1002/cbic.201900596. 31680396

[25] Watts JK, Katolik A, Viladoms J et al. Studies on the hydrolytic stability of 2′-fluoroarabinonucleic acid (2′F-ANA). Org Biomol Chem. 2009; 7:1904–10. 10.1039/b900443b. 19590787

[26] Torres LL, Pinheiro VB Xenobiotic nucleic acid (XNA) synthesis by phi29 DNA polymerase. Curr Protoc Chem Biol. 2018; 10:e41 10.1002/cpch.41. 29927114

[27] Medina E, Yik EJ, Herdewijn P et al. Functional comparison of laboratory-evolved XNA polymerases for synthetic biology. ACS Synth Biol. 2021; 10:1429–37. 10.1021/acssynbio.1c00048. 34029459

[28] Chen T, Hongdilokkul N, Liu Z et al. Evolution of thermophilic DNA polymerases for the recognition and amplification of C2′-modified DNA. Nat Chem. 2016; 8:556–62. 10.1038/nchem.2493. 27219699 PMC4880425

[29] Qin Y, Ma X, Tao R et al. Synthesis, reverse transcription, replication, and inter-transcription of 2′-modified nucleic acids with evolved thermophilic polymerases: efforts toward multidimensional expansion of the central dogma. ACS Synth Biol. 2023; 12:2616–31. 10.1021/acssynbio.3c00213. 37646406

[30] Chen T, Romesberg FE Polymerase chain transcription: exponential synthesis of RNA and modified RNA. J Am Chem Soc. 2017; 139:9949–54. 10.1021/jacs.7b03981. 28715205

[31] Chen T, Romesberg FE Enzymatic synthesis, amplification, and application of DNA with a functionalized backbone. Angew Chem Int Ed Engl. 2017; 56:14046–51. 10.1002/anie.201707367. 28914996

[32] Ng EW, Shima DT, Calias P et al. Pegaptanib, a targeted anti-VEGF aptamer for ocular vascular disease. Nat Rev Drug Discov. 2006; 5:123–32. 10.1038/nrd1955. 16518379

[33] Thirunavukarasu D, Chen T, Liu Z et al. Selection of 2′-fluoro-modified aptamers with optimized properties. J Am Chem Soc. 2017; 139:2892–5. 10.1021/jacs.6b13132. 28218835

[34] Wang Y, Nguyen K, Spitale RC et al. A biologically stable DNAzyme that efficiently silences gene expression in cells. Nat Chem. 2021; 13:319–26. 10.1038/s41557-021-00645-x. 33767363 PMC12582164

[35] Caruthers MH Gene synthesis machines: DNA chemistry and its uses. Science. 1985; 230:281–5. 10.1126/science.3863253. 3863253

[36] Andrews BI, Antia FD, Brueggemeier SB et al. Sustainability challenges and opportunities in oligonucleotide manufacturing. J Org Chem. 2021; 86:49–61. 10.1021/acs.joc.0c02291. 33253568 PMC8154579

[37] Ferrazzano L, Corbisiero D, Tolomelli A et al. From green innovations in oligopeptide to oligonucleotide sustainable synthesis: differences and synergies in TIDES chemistry. Green Chem. 2023; 25:1217–36. 10.1039/D2GC04547H.

[38] Moody ER, Obexer R, Nickl F et al. An enzyme cascade enables production of therapeutic oligonucleotides in a single operation. Science. 2023; 380:1150–4. 10.1126/science.add5892. 37319201

[39] Haslecker R, Pham VV, Glänzer D et al. Extending the toolbox for RNA biology with SegModTeX: a polymerase-driven method for site-specific and segmental labeling of RNA. Nat Commun. 2023; 14:8422 10.1038/s41467-023-44254-3. 38110450 PMC10728113

[40] Van Ness J, Van Ness LK, Galas DJ Isothermal reactions for the amplification of oligonucleotides. Proc Natl Acad Sci USA. 2003; 100:4504–9. 10.1073/pnas.0730811100. 12679520 PMC404692

[41] Ménová P, Hocek M Preparation of short cytosine-modified oligonucleotides by nicking enzyme amplification reaction. Chem Commun. 2012; 48:6921–3. 10.1039/c2cc32930a. 22644213

[42] Ménová P, Raindlová V, Hocek M Scope and limitations of the nicking enzyme amplification reaction for the synthesis of base-modified oligonucleotides and primers for PCR. Bioconjug Chem. 2013; 24:1081–93. 10.1021/bc400149q. 23682869

[43] Brunderová M, Havlíček V, Matyašovský J et al. Expedient production of site specifically nucleobase-labelled or hypermodified RNA with engineered thermophilic DNA polymerases. Nat Commun. 2024; 15:4197 10.1038/s41467-024-48572-y. 38760347 PMC11101615

[44] Taylor AI, Holliger P Selecting fully-modified XNA aptamers using synthetic genetics. Curr Protoc Chem Biol. 2018; 10:e44 10.1002/cpch.44. 29927117

[45] Cozens C, Pinheiro VB XNA synthesis and reverse transcription by engineered thermophilic polymerases. Curr Protoc Chem Biol. 2018; 10:e47 10.1002/cpch.47. 30039931

[46] Yan S, Li X, Zhang P et al. Direct sequencing of 2′-deoxy-2′-fluoroarabinonucleic acid (FANA) using nanopore-induced phase-shift sequencing (NIPSS). Chem Sci. 2019; 10:3110–7. 10.1039/C8SC05228J. 30996894 PMC6429604

[47] Das U, Shuman S Mechanism of RNA 2′,3′-cyclic phosphate end healing by T4 polynucleotide kinase-phosphatase. Nucleic Acids Res. 2013; 41:355–65. 10.1093/nar/gks977. 23118482 PMC3592404

[48] Kalota A, Karabon L, Swider CR et al. 2′-Deoxy-2′-fluoro-β-D-arabinonucleic acid (2′F-ANA) modified oligonucleotides (ON) effect highly efficient, and persistent, gene silencing. Nucleic Acids Res. 2006; 34:451–61. 10.1093/nar/gkj455. 16421272 PMC1342038

[49] Anzahaee MY, Deleavey GF, Le PU et al. Arabinonucleic acids: 2′-stereoisomeric modulators of siRNA activity. Nucleic Acid Ther. 2014; 24:336–43. 10.1089/nat.2014.0496. 25162466

[50] Ferrari N, Bergeron D, TEDESCHI A-L et al. Characterization of antisense oligonucleotides comprising 2′-deoxy-2′-fluoro-β-D-arabinonucleic acid (FANA). Ann N Y Acad Sci. 2006; 1082:91–102. 10.1196/annals.1348.032. 17145930

[51] Ageely EA, Chilamkurthy R, Jana S et al. Gene editing with CRISPR–Cas12a guides possessing ribose-modified pseudoknot handles. Nat Commun. 2021; 12:6591 10.1038/s41467-021-26989-z. 34782635 PMC8593028

[52] Wang Q, Chen L, Long Y et al. Molecular beacons of xeno-nucleic acid for detecting nucleic acid. Theranostics. 2013; 3:395–408. 10.7150/thno.5935. 23781286 PMC3677410

[53] Mana T, Bhattacharya B, Lahiri H et al. XNAs: a troubleshooter for nucleic acid sensing. ACS Omega. 2022; 7:15296–307. 10.1021/acsomega.2c00581. 35571783 PMC9096816

[54] Simeonov A, Nikiforov TT Single nucleotide polymorphism genotyping using short, fluorescently labeled locked nucleic acid (LNA) probes and fluorescence polarization detection. Nucleic Acids Res. 2002; 30:e91 10.1093/nar/gnf090. 12202779 PMC137436

[55] Hu J, Xiao K, Jin B et al. Paper-based point-of-care test with xeno nucleic acid probes. Biotechnol Bioeng. 2019; 116:2764–77. 10.1002/bit.27106. 31282991

[56] Sun L, Xiang Y, Du Y et al. Template-independent synthesis and 3′-end labelling of 2′-modified oligonucleotides with terminal deoxynucleotidyl transferases. Nucleic Acids Res. 2024; 52:10085–101. 10.1093/nar/gkae691. 39149896 PMC11417362

[57] Hocek M Synthesis of base-modified 2′-deoxyribonucleoside triphosphates and their use in enzymatic synthesis of modified DNA for applications in bioanalysis and chemical biology. J Org Chem. 2014; 79:9914–21. 10.1021/jo5020799. 25321948

[58] Liu Y, Holmstrom E, Zhang J et al. Synthesis and applications of RNAs with position-selective labelling and mosaic composition. Nature. 2015; 522:368–72. 10.1038/nature14352. 25938715 PMC4800989

[59] Wang G, He C, Zou J et al. Enzymatic synthesis of DNA with an expanded genetic alphabet using terminal deoxynucleotidyl transferase. ACS Synth Biol. 2022; 11:4142–55. 10.1021/acssynbio.2c00456. 36455255

