# Supplementary material for: A solid-phase enzymatic synthesis platform for the facile production of 2′-fluoroarabinonucleic acid (FANA) and chimeric XNA oligonucleotides using an evolved XNA polymerase
Source: Nucleic Acids Res. 2025 Jun 26;53(12):gkaf567. doi: 10.1093/nar/gkaf567 (PMC12199150; doi:10.1093/nar/gkaf567)
Supplement: gkaf567_Supplemental_File [file gkaf567_supplemental_file.pdf]

Supplementary Data

A Solid-Phase Enzymatic Synthesis Platform for the Facile Production of 2'-Fluoroarabinonucleic Acid (FANA) and Chimeric XNA Oligonucleotides Using an Evolved XNA Polymerase

Binliang Zhang[a, ‡], Yuhui Du[a, ‡], Jingxing Zhang[a], Xingyun Ma[a], Yanjia Qin[a], Rui Tao[a], Minglan Luo[a], Jing Wu[a], Leping Sun[a], Gan Zhu[a], Hantao Luo[a], Junlin Wen[b], Chenghe Xiong[b], Hui Mei[b,*] and Tingjian Chen[a,*]

[a]MOE International Joint Research Laboratory on Synthetic Biology and Medicines, School of Biology and Biological Engineering, South China University of Technology, Guangzhou 510006, China.

[b]Shenzhen Key Laboratory of Synthetic Genomics, Guangdong Provincial Key Laboratory of Synthetic Genomics, State Key Laboratory of Quantitative Synthetic Biology, Shenzhen Institute of Synthetic Biology, Shenzhen Institutes of Advanced Technology, Chinese Academy of Sciences, Shenzhen 518055, China

*To whom correspondence should be addressed. Email: chentj@scut.edu.cn.

Correspondence may also be addressed to Hui Mei. Email: hui.mei@siat.ac.cn.

[‡]The first two authors should be regarded as Joint First Authors.

Supplementary Table

Table S1. Oligonucleotides used in this study

Name	Sequence (5′-3′)
FAM-P20	FAM-GGCTTTACACTTTATGACGG
T50	TCTCCGCCCGATCCCTCTCCACACTGATTACCGTCATAAAGTGTAAAGCC
T70	GTCTGAGTCTCATGTACTGGTCTCCGCCCGATCCCTCTCCACACTGATTACCGTCATAAAG TGTAAAGCC
Cy3-P20	Cy3-CATAGTCTAGAGATTGAGTA
P20-RT	CATAGTCTAGAGATTGAGTA
T-RT68	CATAGTCTAGAGATTGAGTAACTTCAGTCTGGACAGTGATCGCTCCAGGAAGTACATGAG ACTCAGAC
B-P18	Biotin-GTCTGAGTCTCATGTACT
EcoRI-F	GATCCGAATTCGCATAGTCTAGAGATTGAGTA
HindIII-R	CCGCAAGCTTGTCTGAGTCTCATGTACT
Cy3-P22	Cy3-GGCTTCGTATGTTGTGTGGACT
T-A	GCAATAGTCCACACAACATACGAAGCC
T-U	GCTTAAGTCCACACAACATACGAAGCC
T-G	TAGGCAGTCCACACAACATACGAAGCC
T-C	TACCGAGTCCACACAACATACGAAGCC
T-rA	CGGGTGACTAGATCCGTCATAAAGTGTAAAGCC
T-rU	CGGGTGACTAGTACCGTCATAAAGTGTAAAGCC
T-rG	CGGGTGACTAGGCCCGTCATAAAGTGTAAAGCC
T-rC	CGGGTGACTAGCGCCGTCATAAAGTGTAAAGCC
T-rAEdU	CGGGTGACTAGCATCCGTCATAAAGTGTAAAGCC
T-rAC^{Am}	CGGGTGACTAGCGTCCGTCATAAAGTGTAAAGCC
T-rA-UC	CGGGTGACTAGCGATCCGTCATAAAGTGTAAAGCC
T-55U	N_3-TCACATCCGGAAGTCAAACCGGGTGACTAGTACACGTGCACCATTGGTGCACGTG
T-55A	N_3-TCACATCCGGAAGTCAAACCGGGTGACTAGATCACGTGCACCATTGGTGCACGTG
T-55G	N_3-TCACATCCGGAAGTCAAACCGGGTGACTAGGCCACGTGCACCATTGGTGCACGTG

T-55C	N_3-TCACATCCGGAAGTCAAACCGGGTGACTAGCGCACGTGCACCATTGGTGCACGTG
B-T-55U	Biotin-TCACATCCGGAAGTCAAACCGGGTGACTAGTACACGTGCACCATTGGTGCACGTG
T-56-rAU	N_3-TCACATCCGGAAGTCAAACCGGGTGACTAGCATCACGTGCACCATTGGTGCACGTG
T-56-rAC	N_3-TCACATCCGGAAGTCAAACCGGGTGACTAGCGTCACGTGCACCATTGGTGCACGTG
T-57-rAUC	N_3-TCACATCCGGAAGTCAAACCGGGTGACTAGCGATCACGTGCACCATTGGTGCACGTG
P20	GGCTTTACACTTTATGACGG
FR6_1_BR af600	faAfaCfaUfaAfaGfaUfaCfaAfaCfaCfaCfaGfaGfaUfaUfaUfaGfaAfaCfaUfaUfaCfaCfaGfaGfaAfaU faGfaUfaGfaA
Cy3-R30	Cy3-rGrGrUrCrArCrArUrCrArGrArGrArCrUrArGrCrArUrCrGrArUrUrCrCrUrU

*fa: 2'-deoxy-2'-fluoroarabinonucleotide; r: ribonucleotide.

Table S2. XNA polymerases derived from family A and B DNA polymerases

Family	Polymerase	Mutations	Function	Ref.
A	SFM4-3	SFM19: V518A, N583S, D655N, E681K, E742Q, M747R	Synthesis or amplification of 2'-OMe, 2'-F, 2'-Az, 2'-Cl and 2'-Am-modified nucleic acids, RNA, and ANA	1, 2
	SFM4-6	SFM19: D655N, L657M, E681K, E742N, M747R	Synthesis of 2'-OMe-RNA	1
	SFM4-9	SFM19: N415Y, V518A, D655N, L657M, E681V, E742N, M747R	Reverse transcription of 2'-OMe-RNA	1
	SFM5-7	SFM4-3: E520P, E681R	Synthesis of 2'-OMe-RNA, 2'-F-RNA and RNA; reverse transcription of 2'-F-RNA, 2'-OMe-RNA and RNA; replication of 2'-OMe-RNA, 2'-F-RNA and RNA; inter-transcription of 2'-OMe-RNA, 2'-F-RNA and RNA	3
B	Deep Vent DNAP	-	Synthesis of **FANA**	4
	Tgo DNAP	-	Synthesis of **FANA**	4, 5
	Tgo Pol6G12	TgoT: V589A, E609K, I610M, K659Q, E664Q, Q665P, R668K, D669Q, K671H, K674R, T676R, A681S, L704P, E730G	Synthesis of HNA and **FANA**	4, 6
	Tgo-6G12-I521L	Pol6G12: I521L	Synthesis of HNA, ANA and **FANA**	4
	Tgo RT521	TgoT: E429G, I521L, K726R	Synthesis of TNA<br>Reverse transcription of HNA, ANA, **FANA**, TNA and tPhoNA	6, 7
	Tgo-D4K	TgoT: L403P, P657T, E658Q, K659H, Y663H, E664K, D669A, K671N, T676I	Synthesis of **FANA**, ANA, TNA, HNA and RNA	4, 6
	Tgo QGLK	Tgo: V93Q, D141A, E143A, Y409G, A485L, E664K	Synthesis of RNA, **FANA** and ANA	4, 8
	TgoT-EPFLH	TgoT: H147E, L403P, L408F, I521L, E664H	Synthesis of PMT, HNA, ANA, TNA, RNA, **FANA** and tPhoNA	4, 7

Table S2 (Continued). XNA polymerases derived from family A and B DNA polymerases

Family	Polymerase	Mutations	Function	Ref.
B	Kod DNAP	-	Synthesis of **FANA**	4
	Kod-RSGA	Kod RS: R606G, T723A	Synthesis of **FANA**, ANA, TNA and C5-modified TNA	4, 9, 10
	9°N DNAP	-	Synthesis of **FANA**	4
	Phi29 DNAP mutant	Phi29 DNAP: D12A	Synthesis of HNA, **FANA** and 2'-F-RNA	11

Supplementary Figures

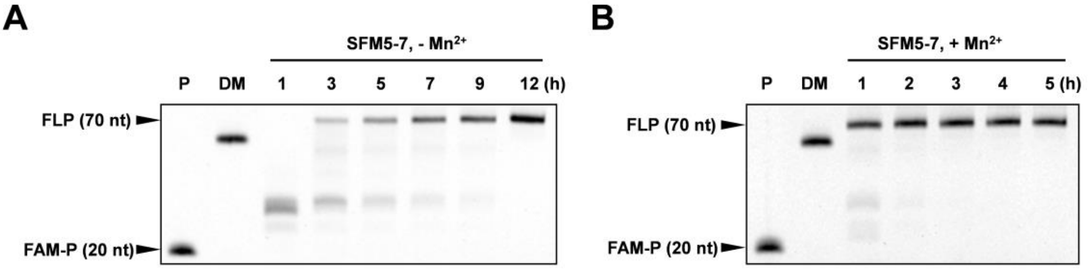

Figure S1. Time courses of FANA synthesis mediated by SFM5-7. (**A**) Time course of FANA synthesis mediated by SFM5-7 in the absence of Mn^{2+}. Primer extension reaction was carried out by mixing 100 nM 5'-FAM-labeled DNA primer FAM-P20/DNA template T70, 5 mM $MgCl_2$, 0.4 mM each of faNTPs, and 2.5 μM SFM5-7 in 1× standard Taq reaction buffer and incubating the mixture at 50 °C for 1-12 h. The products were analyzed with denaturing PAGE. (**B**) Time course of FANA synthesis mediated by SFM5-7 in the presence of 1 mM Mn^{2+}. Primer extension reaction was carried by mixing 100 nM 5'-FAM-labeled DNA primer FAM-P20/DNA template T70, 5 mM $MgCl_2$, 1 mM $MnCl_2$, 0.4 mM each of faNTPs, and 2.5 μM SFM5-7 in 1× standard Taq reaction buffer and incubating the mixture at 50 °C for 1-5 h. FLP: full-length product; FAM-P/P: FAM-labeled primer FAM-P20 (20 nt); DM: DNA size marker for the full-length extension product, produced by extending primer FAM-P20 with dNTPs, template T70 and SFM5-7.

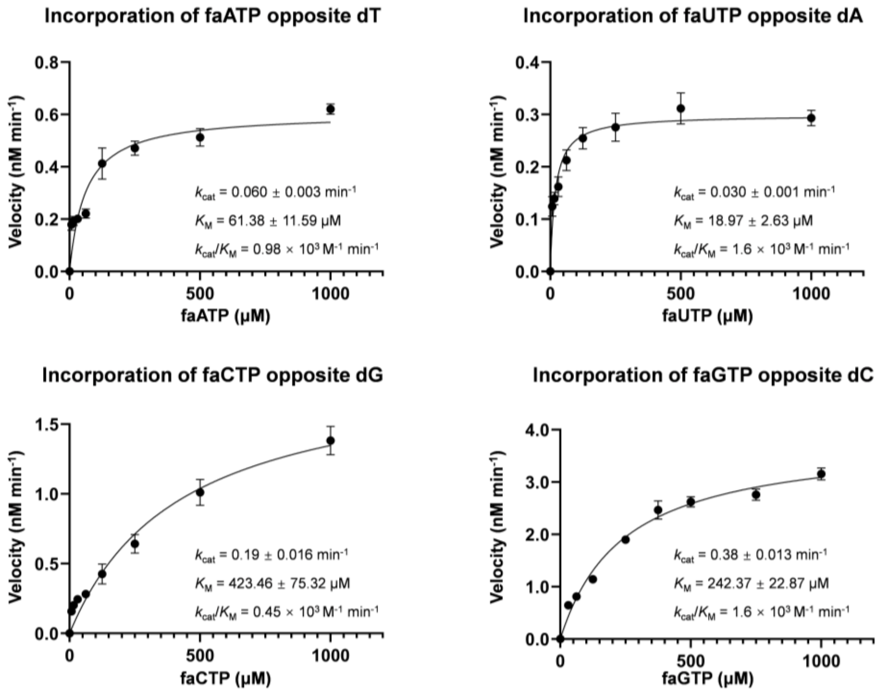

Figure S2. Michaelis-Menten plots for SF WT-mediated incorporation of faATP, faUTP, faCTP and faGTP opposite dT, dA, dG and dC, respectively, in the DNA template.

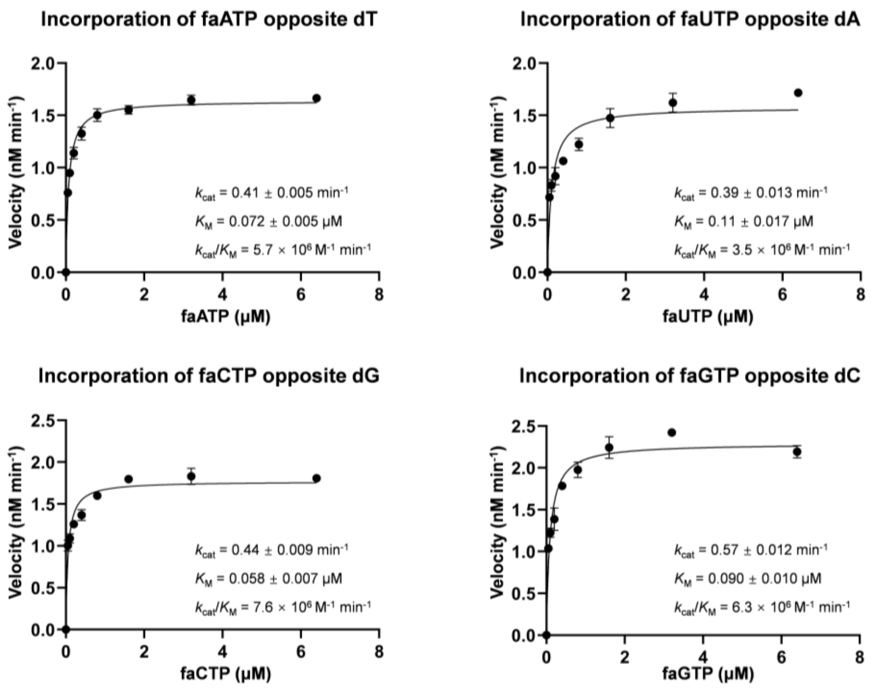

Figure S3. Michaelis-Menten plots for SFM5-7-mediated incorporation of faATP, faUTP, faCTP and faGTP opposite dT, dA, dG and dC, respectively, in the DNA template.

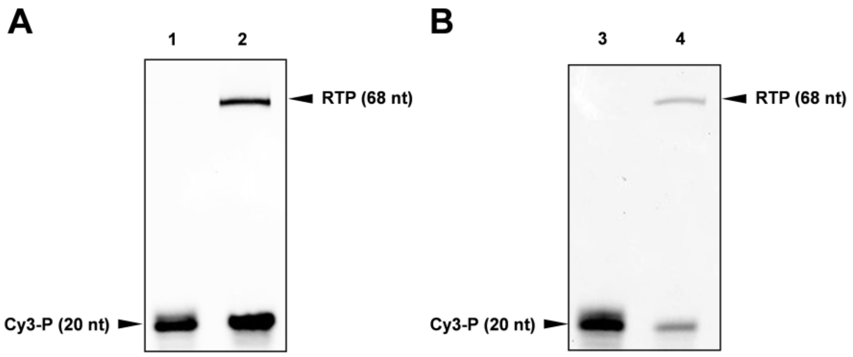

Figure S4. Reverse transcription of FANA into DNA by phi29 DNA polymerase. (**A**) Denaturing PAGE analysis of the product of phi29 DNA polymerase-mediated reverse transcription of FANA (prepared with SFM5-7 in the absence of Mn^{2+}). The DNA-FANA template was prepared by primer extension via FANA synthesis with a biotinylated DNA primer, B-P18 (Table S1), and SFM5-7 in the absence of Mn^{2+}, purified with SA-coated magnetic beads, and then used for phi29 DNA polymerase-mediated reverse transcription. Lane 1: primer for reverse transcription, Cy3-P20 (20 nt); lane 2: reverse transcription product. (**B**) Denaturing PAGE analysis of the product of phi29 DNA polymerase-mediated reverse transcription of FANA (prepared with SFM5-7 in the presence of 1 mM Mn^{2+}). The DNA-FANA template was prepared by primer extension via FANA synthesis with biotinylated DNA primer B-P18 and SFM5-7 in the presence of 1 mM Mn^{2+}, purified with SA-coated magnetic beads, and then used for phi29 DNA polymerase-mediated reverse transcription. Lane 3: primer for reverse transcription, Cy3-P20 (20 nt); lane 4: reverse transcription product. Cy3-P: primer for reverse transcription, Cy3-P20 (20 nt); RTP: reverse transcription product.

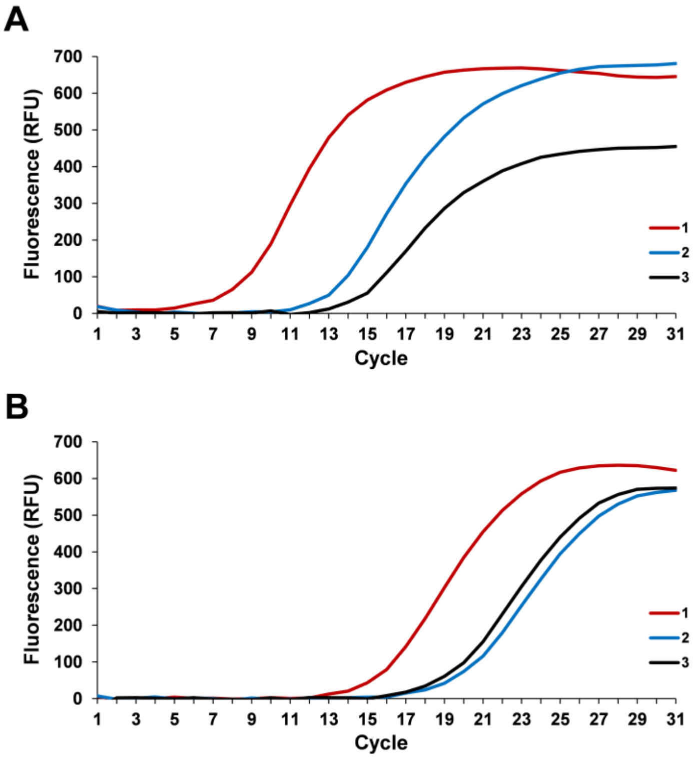

Figure S5. qPCR assays for the integrated fidelity tests of FANA transcription-reverse transcription-PCR. FANA was transcribed with a biotinylated primer and SFM5-7 in the absence of Mn^{2+} (**A**) or in the presence of 1 mM Mn^{2+} (**B**). 1: the transcription product produced by SFM5-7 was purified with streptavidin-coated magnetic beads, during which the original DNA template was removed by NaOH washing, and reverse transcribed back into DNA using phi29 DNA polymerase, and then the reverse transcription product was subjected to qPCR assay; 2: same as 1, except that phi29 DNAP was excluded in the reaction solution for reverse transcription; 3: control experiment, where no reverse transcription product was included in the reaction solution for qPCR assay.

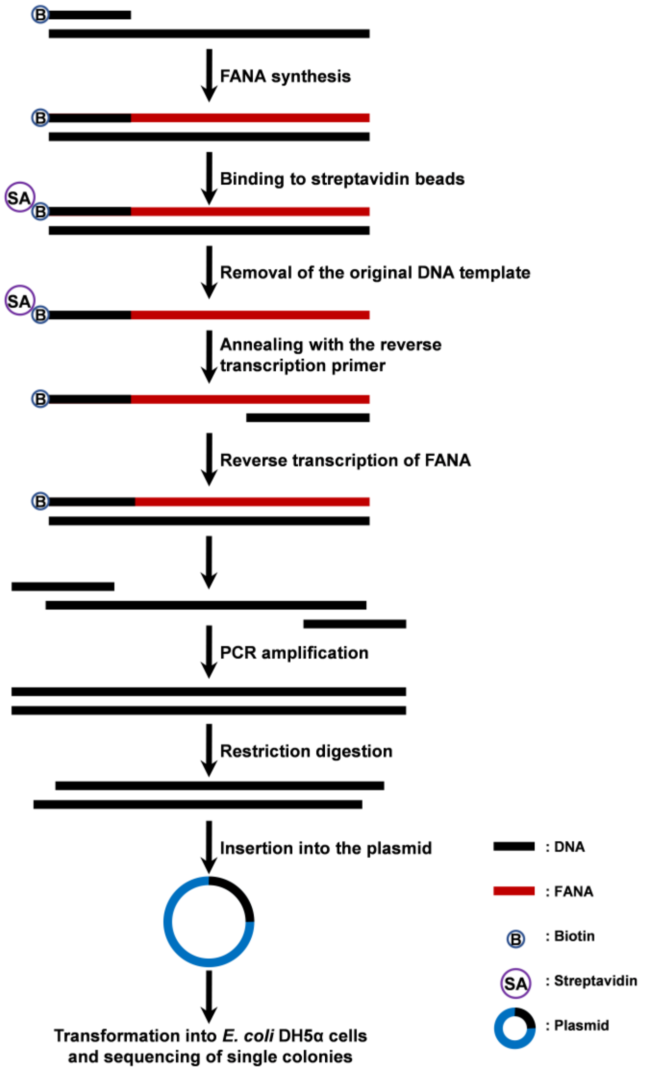

Figure S6. Scheme for the fidelity test of SFM5-7-mediated FANA synthesis.

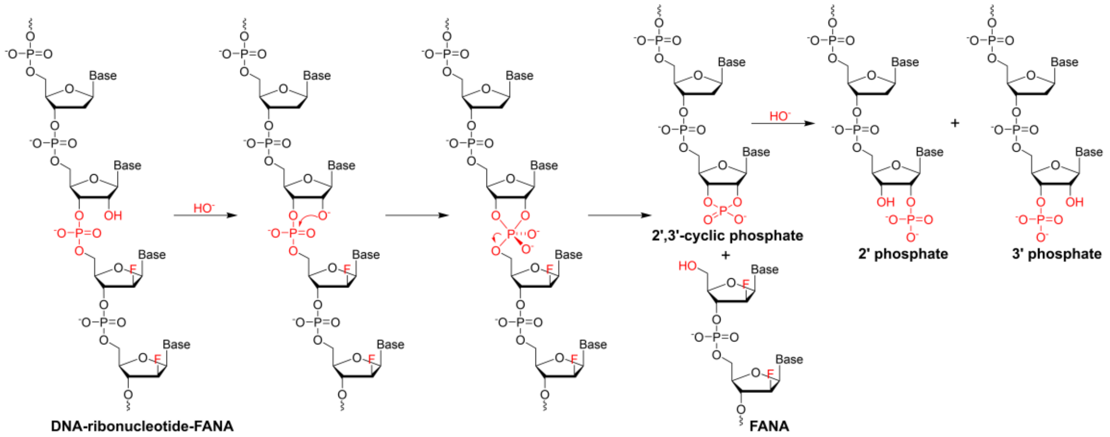

Figure S7. Mechanism for the alkaline cleavage of DNA-ribonucleotide-FANA. The 2' hydroxyl group of the ribonucleotide is deprotonated under alkaline conditions, leading to a nucleophilic attack on the 5' phosphate of the adjacent FANA nucleotide. After the leaving of the FANA oligonucleotide, the generated 2',3'-cyclic phosphate can be further hydrolyzed to form a 2' or 3' phosphate on the ribonucleotide.

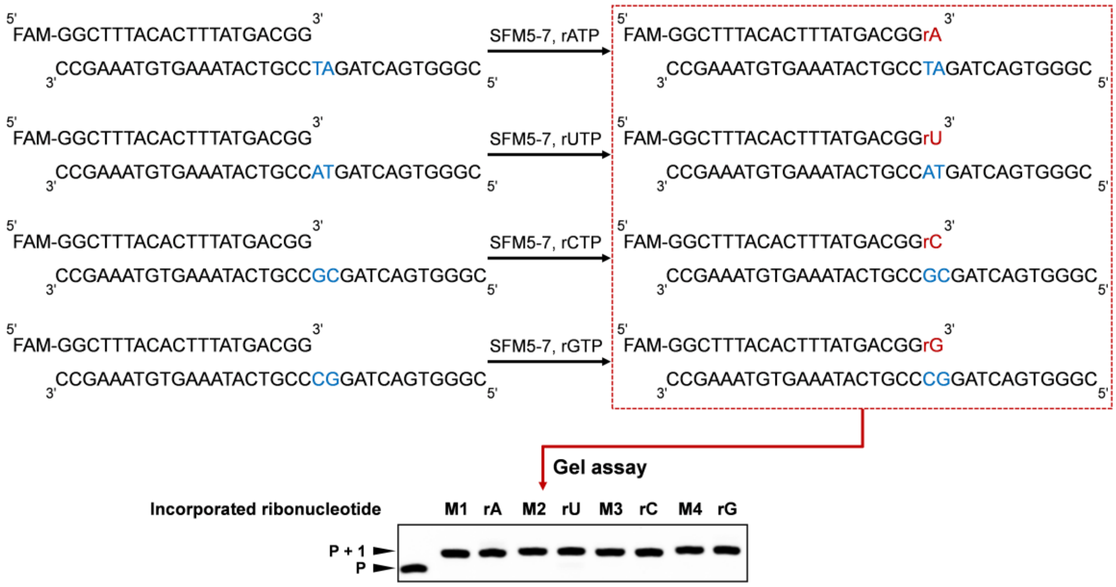

Figure S8. Assay for the incorporation of a single ribonucleotide opposite the corresponding deoxyribonucleotide in different templates by SFM5-7. FAM-labeled primer FAM-P20 was annealed with template T-rA, T-rU, T-rC or T-rG in 1× standard Taq reaction buffer by incubating the solution at 95 °C for 10 min and slowly cooling it down to room temperature. Then 0.2 µM of the primer/template was mixed with 1 µM of the corresponding rNTP and 1 µM SFM5-7 in 1× standard Taq reaction buffer and incubated at 50 °C for 10 min. The reactions were quenched by the addition of 2× TBE-urea sample buffer and the resultant solutions were incubated at 95 °C for 10 min. The products were analyzed with a 20% denaturing PAGE gel containing 8 M urea. P: primer FAM-P20; P + 1: FAM-P20 incorporating a ribonucleotide at its 3' end. M1-M4: size makers for the products (FAM-P20 incorporating rA, FAM-P20 incorporating rU, FAM-P20 incorporating rC and FAM-P20 incorporating rG), which were prepared with the following protocol. 5'-FAM-labeled DNA primer FAM-P20 was extended by mixing 0.2 µM FAM-P20/DNA template T-rA, T-rU, T-rC or T-rG, with 2 mM rATP, rUTP, rCTP or rGTP, and 5 µM SFM5-7 in 1× standard Taq reaction buffer and incubating the mixture at 50 °C for 30 min. The extension product was mixed with 500 mM NaOH and incubated at 65 °C for 20 min. The NaOH cleavage product was neutralized with HCl, purified with a Zymo ssDNA/RNA Clean & Concentrator™ kit. Then the NaOH cleavage product was mixed with 5 U/µL T4 PNK in 1× T4 PNK buffer

and incubated at 37 °C for 12 h to dephosphorylate the 3' end ribonucleotide, resulting in the production of primer FAM-P20 incorporating an rA, rU, rC or rG nucleotide.

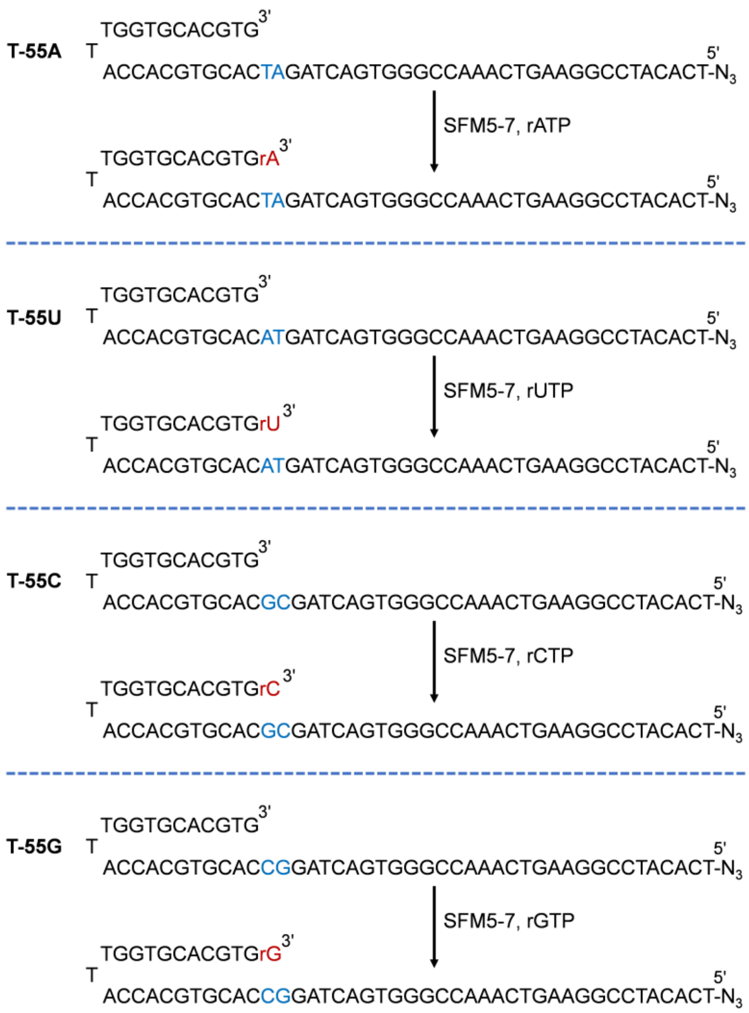

Figure S9. Design of four self-priming hairpin DNA templates for the production of FANA and other XNA oligonucleotides with different 5'-end nucleotides. Self-priming hairpin DNA templates T-55A, T-55U, T-55C and T-55G were designed and synthesized, and each of them has a dT, dA, dG or dC deoxyribonucleotide at the position opposite the position where an rU, rA, rC or rG ribonucleotide would be incorporated to extend the 3' end of the folded self-priming hairpin DNA template, and a different 5' deoxyribonucleotide adjacent to this deoxyribonucleotide. Each of these four self-priming hairpin DNA templates could be extended with SFM5-7 and the corresponding rNTP to incorporate a single ribonucleotide into its 3' end.

Oligonucleotide	FANA
Components	faA, faC, faU, faG
Amount of the template	100.0 pmol
Amount of the product	56.3 pmol
Yield	56.3%
Purity	> 95%
Expected mass	9934.3
Observed mass	9935.0
Incorporation of non-templated nucleotides at the 3' end	No

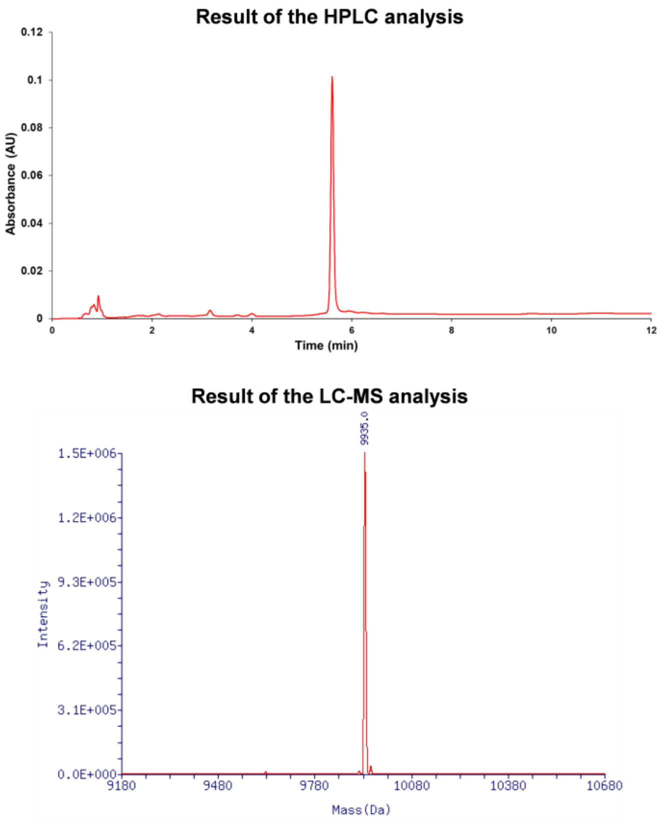

Figure S10. High performance liquid chromatography (HPLC) and liquid chromatography-mass spectrometry (LC-MS) characterization of the FANA oligonucleotide prepared by the SPEXOS platform. The amount of the product was calculated from the band intensities of the products quantified from the gel images, using a chemically synthesized oligonucleotide with a known concentration as the standard.

Oligonucleotide	2'-F-RNA
Components	2'-F-A, 2'-F-C, 2'-F-U, 2'-F-G
Amount of the template	100.0 pmol
Amount of the product	68.7 pmol
Yield	68.7%
Purity	> 95%
Expected mass	9934.3
Observed mass	10267.0 (M + a 2'-F-A nucleotide)
Incorporation of non-templated nucleotides at the 3' end	Yes. A 2'-F-A nucleotide was incorporated

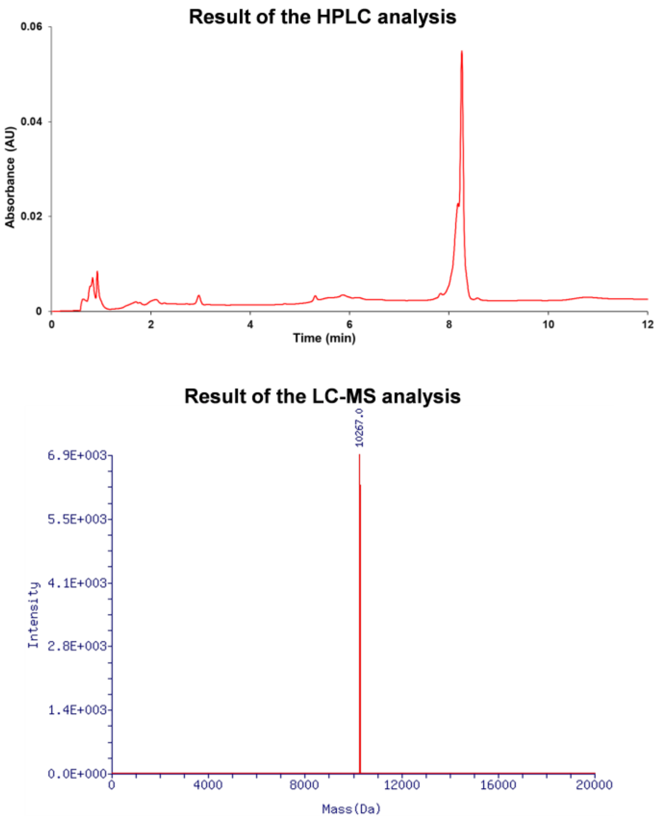

Figure S11. HPLC and LC-MS characterization of the 2'-F-RNA oligonucleotide prepared by the SPEXOS platform. The amount of the product was calculated from the band intensities of the products quantified from the gel images, using a chemically synthesized oligonucleotide with a known concentration as the standard.

Oligonucleotide	2'-OMe-RNA
Components	2'-OMe-A, 2'-OMe-C, 2'-OMe-U, 2'-OMe-G
Amount of the template	100.0 pmol
Amount of the product	46.8 pmol
Yield	46.8%
Purity	> 95%
Expected mass	10305.4
Observed mass	10308.1, 10329.8 (M + Na^+), 10351.1 (M + $2Na^+$)
Incorporation of non-templated nucleotides at the 3' end	No

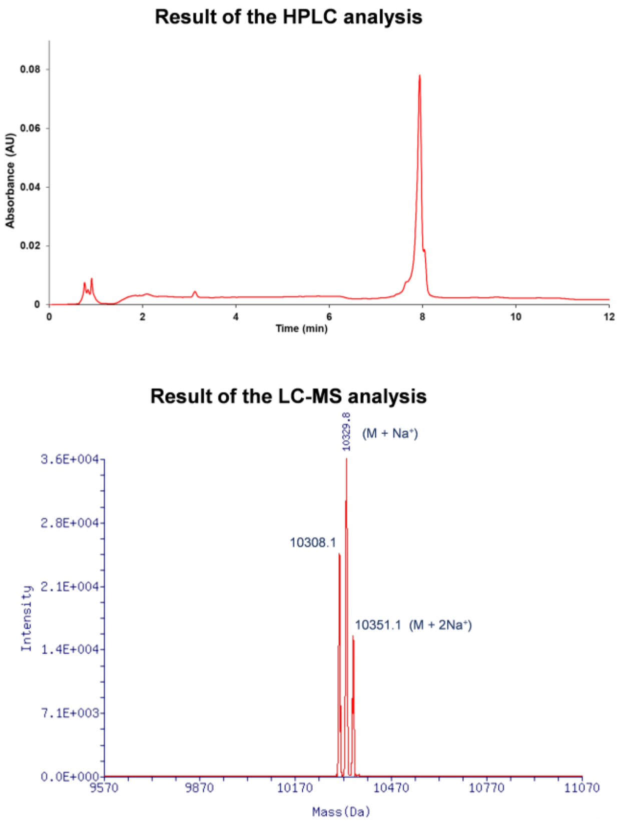

Figure S12. HPLC and LC-MS characterization of the 2'-OMe-RNA oligonucleotide prepared by the SPEXOS platform. The amount of the product was calculated from the band intensities of the products quantified from the gel images, using a chemically synthesized oligonucleotide with a known concentration as the standard.

Oligonucleotide	Chimeric 2'-F-RNA/ 2'-OMe-RNA
Components	2'-F-C, 2'-F-U; 2'-OMe-A, 2'-OMe-G
Amount of the template	100.0 pmol
Amount of the product	61.0 pmol
Yield	61.0%
Purity	> 95%
Expected mass	10102.3
Observed mass	10104.3
Incorporation of non-templated nucleotides at the 3' end	No

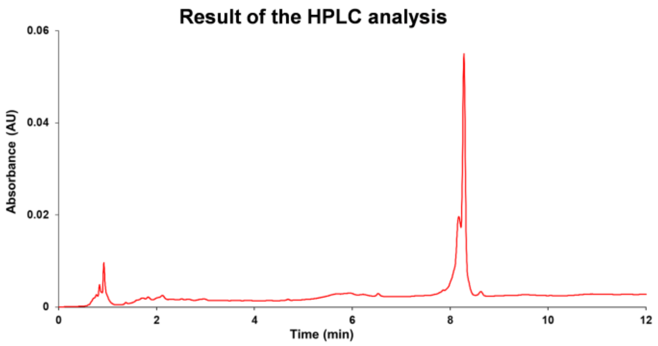

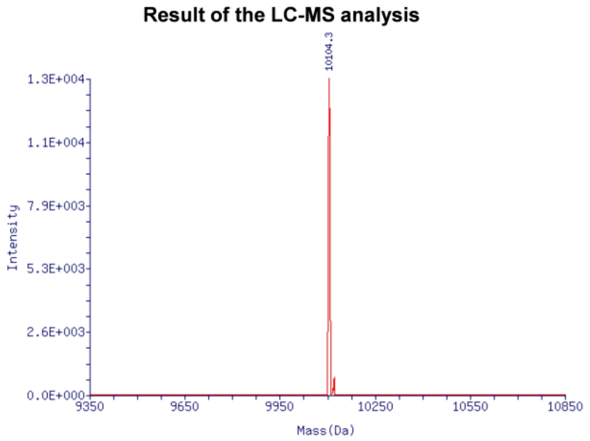

Figure S13. HPLC and LC-MS characterization of the chimeric 2'-F-RNA/2'-OMe-RNA oligonucleotide prepared by the SPEXOS platform. The amount of the product was calculated from the band intensities of the products quantified from the gel images, using a chemically synthesized oligonucleotide with a known concentration as the standard.

Oligonucleotide	Chimeric FANA/ DNA
Components	faA, faG; dC, dT
Amount of the template	100.0 pmol
Amount of the product	63.6 pmol
Yield	63.6%
Purity	> 95%
Expected mass	9754.0
Observed mass	9755.3
Incorporation of non-templated nucleotides at the 3' end	No

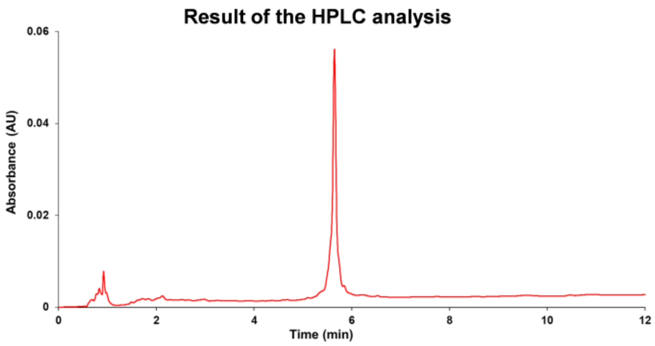

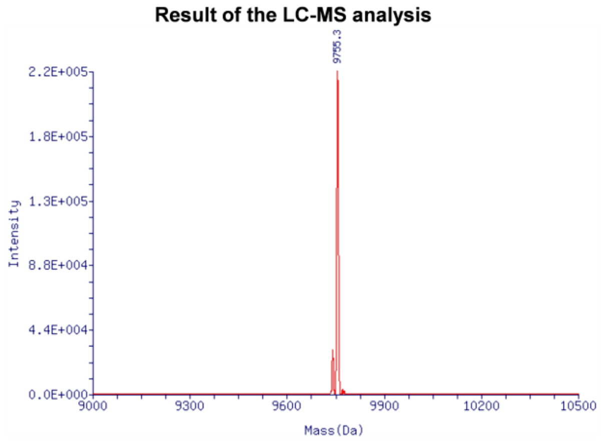

Figure S14. HPLC and LC-MS characterization of the chimeric FANA/DNA oligonucleotide prepared by the SPEXOS platform. The amount of the product was calculated from the band intensities of the products quantified from the gel images, using a chemically synthesized oligonucleotide with a known concentration as the standard.

Oligonucleotide	Chimeric FANA/ 2'-F-RNA
Components	faA, faG; 2'-F-C, 2'-F-U
Amount of the template	100.0 pmol
Amount of the product	66.3 pmol
Yield	66.3%
Purity	> 95%
Expected mass	9934.3
Observed mass	9936.8
Incorporation of non-templated nucleotides at the 3' end	No

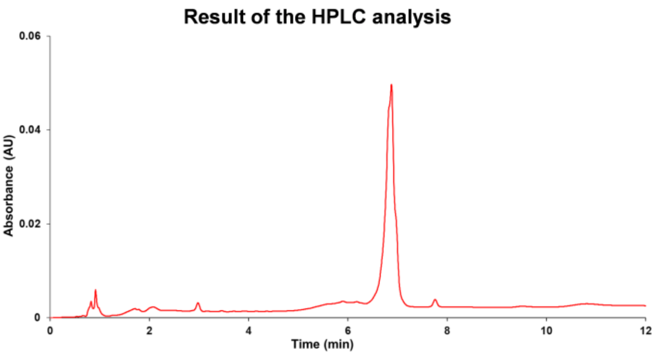

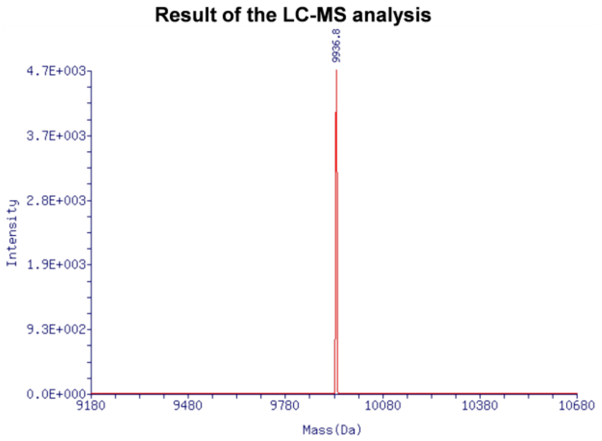

Figure S15. HPLC and LC-MS characterization of the chimeric FANA/2'-F-RNA oligonucleotide prepared by the SPEXOS platform. The amount of the product was calculated from the band intensities of the products quantified from the gel images, using a chemically synthesized oligonucleotide with a known concentration as the standard.

Oligonucleotide	Chimeric FANA/ 2'-F-RNA/ 2'-OMe-RNA
Components	faG; 2'-F-C, 2'-F-U; 2'-OMe-A
Amount of the template	100.0 pmol
Amount of the product	58.0 pmol
Yield	58.0%
Purity	> 95%
Expected mass	10006.3
Observed mass	10006.8
Incorporation of non-templated nucleotides at the 3' end	No

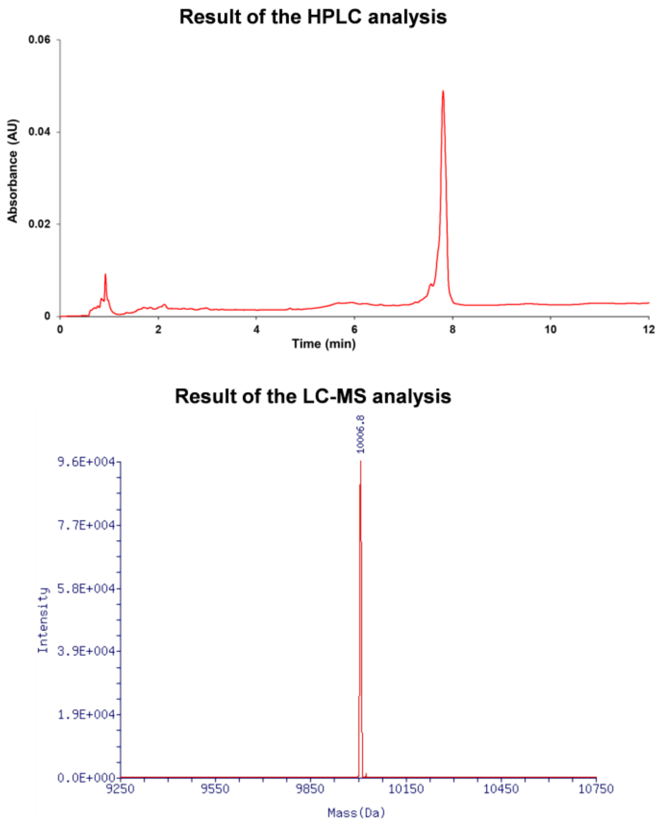

Figure S16. HPLC and LC-MS characterization of the chimeric FANA/2'-F-RNA/2'-OMe-RNA oligonucleotide prepared by the SPEXOS platform. The amount of the product was calculated from the band intensities of the products quantified from the gel images, using a chemically synthesized oligonucleotide with a known concentration as the standard.

Oligonucleotide	Chimeric FANA/DNA/ 2'-OMe-RNA
Components	faG; dC, dT; 2'-OMe-A
Amount of the template	100.0 pmol
Amount of the product	53.9 pmol
Yield	53.9%
Purity	> 95%
Expected mass	9826.0
Observed mass	9826.0
Incorporation of non-templated nucleotides at the 3' end	No

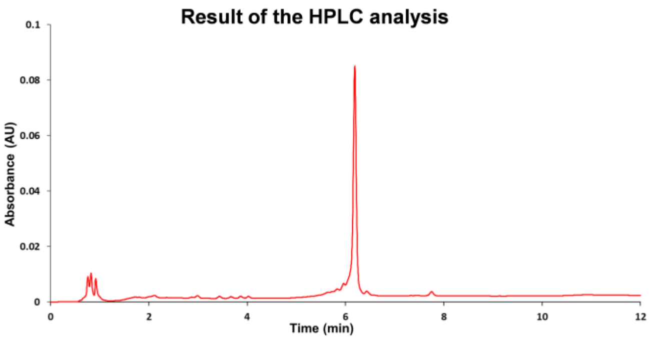

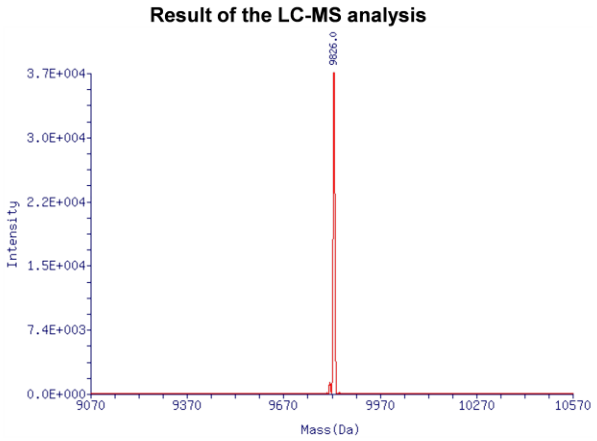

Figure S17. HPLC and LC-MS characterization of the chimeric FANA/DNA/2'-OMe-RNA oligonucleotide prepared by the SPEXOS platform. The amount of the product was calculated from the band intensities of the products quantified from the gel images, using a chemically synthesized oligonucleotide with a known concentration as the standard.

**Oligonucleotide**	**Chimeric FANA/ DNA/2'-F-RNA /2'-OMe-RNA**
**Components**	**faG; dC; 2'-F-U; 2'-OMe-A**
**Amount of the template**	**100.0 pmol**
**Amount of the product**	**59.2 pmol**
**Yield**	**59.2%**
**Purity**	**> 95%**
**Expected mass**	**9862.1**
**Observed mass**	**9862.9**
**Incorporation of non-templated nucleotides at the 3' end**	**No**

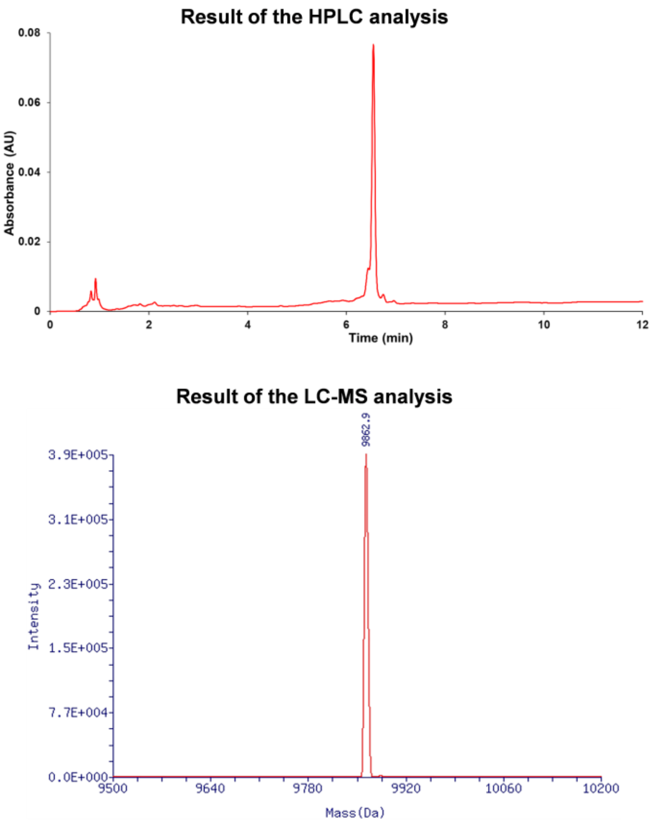

Figure S18. HPLC and LC-MS characterization of the chimeric FANA/DNA/2'-F-RNA/2'-OMe-RNA oligonucleotide prepared by the SPEXOS platform. The amount of the product was calculated from the band intensities of the products quantified from the gel images, using a chemically synthesized oligonucleotide with a known concentration as the standard.

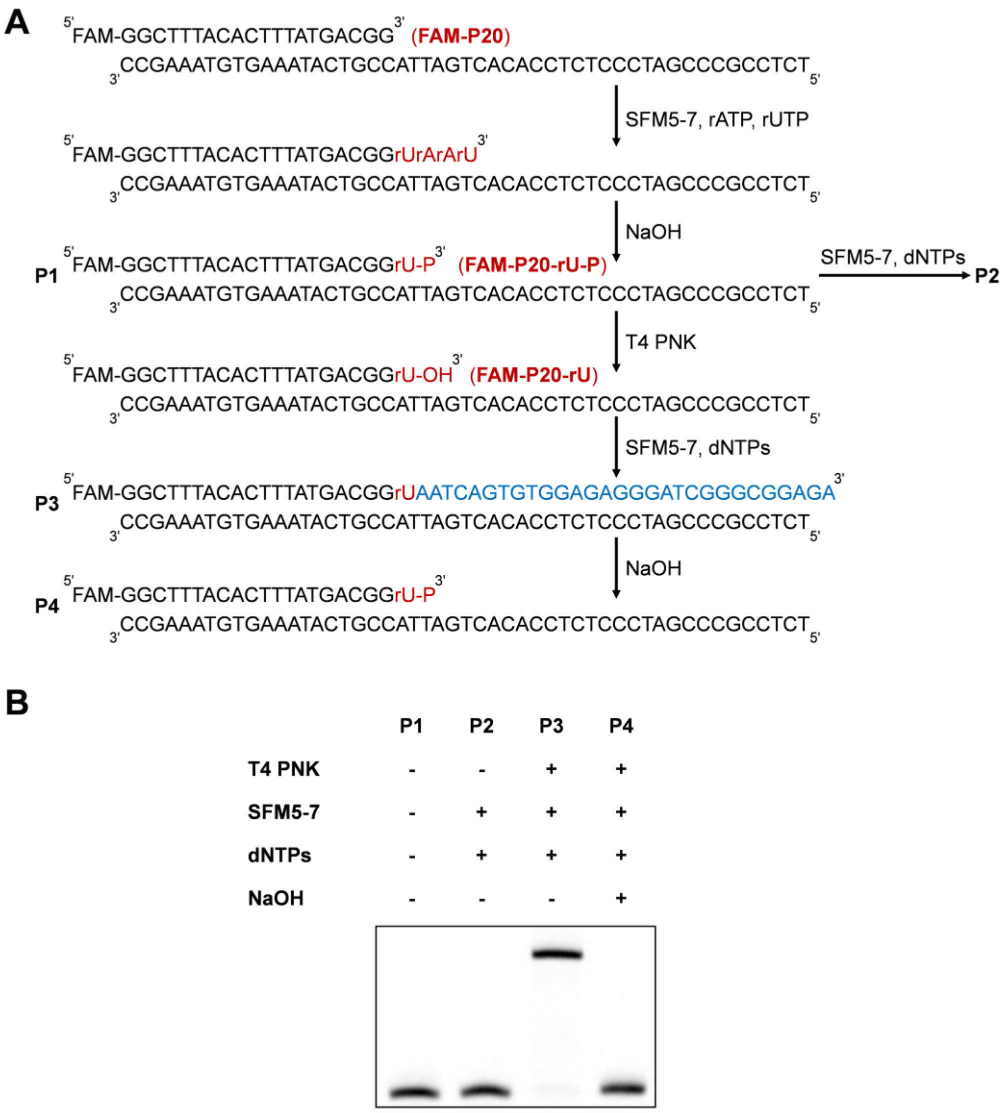

Figure S19. Validation of the strategy of using T4 PNK to regenerate the self-priming hairpin DNA template after NaOH cleavage of the oligonucleotide product. (**A**) Scheme of the experiment. 5'-FAM-labeled DNA primer FAM-P20 was extended by mixing 0.2 μM FAM-P20/DNA template T50 with 2 mM rATP, 2 mM rUTP and 5 μM SFM5-7 in 1× standard Taq reaction buffer and incubating the mixture at 50 °C for 30 min. The extension product was mixed with 500 mM NaOH and incubated at 65 °C for 20 min to produce a DNA primer with a 2' or 3' phosphate on the 3'-end ribonucleotide (FAM-P20-rU-P). The NaOH cleavage product was neutralized with HCl, purified with a Zymo ssDNA/RNA Clean & Concentrator™ kit. Then 500 nM FAM-P20-rU-P/T50 was mixed with 5 U/μL T4 PNK in 1× T4 PNK buffer and incubated at 37 °C for 12 h

to dephosphorylate the 3' end ribonucleotide, resulting in the production of primer FAM-P20-rU. Next, primer FAM-P20-rU was extended by mixing 100 nM FAM-P20-rU/DNA template T50 with 0.5 mM each of dNTPs and 5 μM SFM5-7 in 1× standard Taq reaction buffer and incubating the mixture at 50 °C for 30 min. For comparison, primer FAM-P20-rU-P was also extended with the same reaction conditions. Then the cleavability of the extension product was verified by incubating the extension product of FAM-P20-rU with 500 mM NaOH at 65 °C for 20 min. The NaOH cleavage product was neutralized with HCl before gel analysis. Products P1, P2, P3 and P4 in the scheme were analyzed with denaturing PAGE (**B**). Lane P1: FAM-P20-rU-P/T50; lane P2: product of extending FAM-P20-rU-P with dNTPs and SFM5-7; lane P3: product of extending FAM-P20-rU with dNTPs and SFM5-7; lane P4: NaOH-cleaved product of extending FAM-P20-rU with dNTPs and SFM5-7.

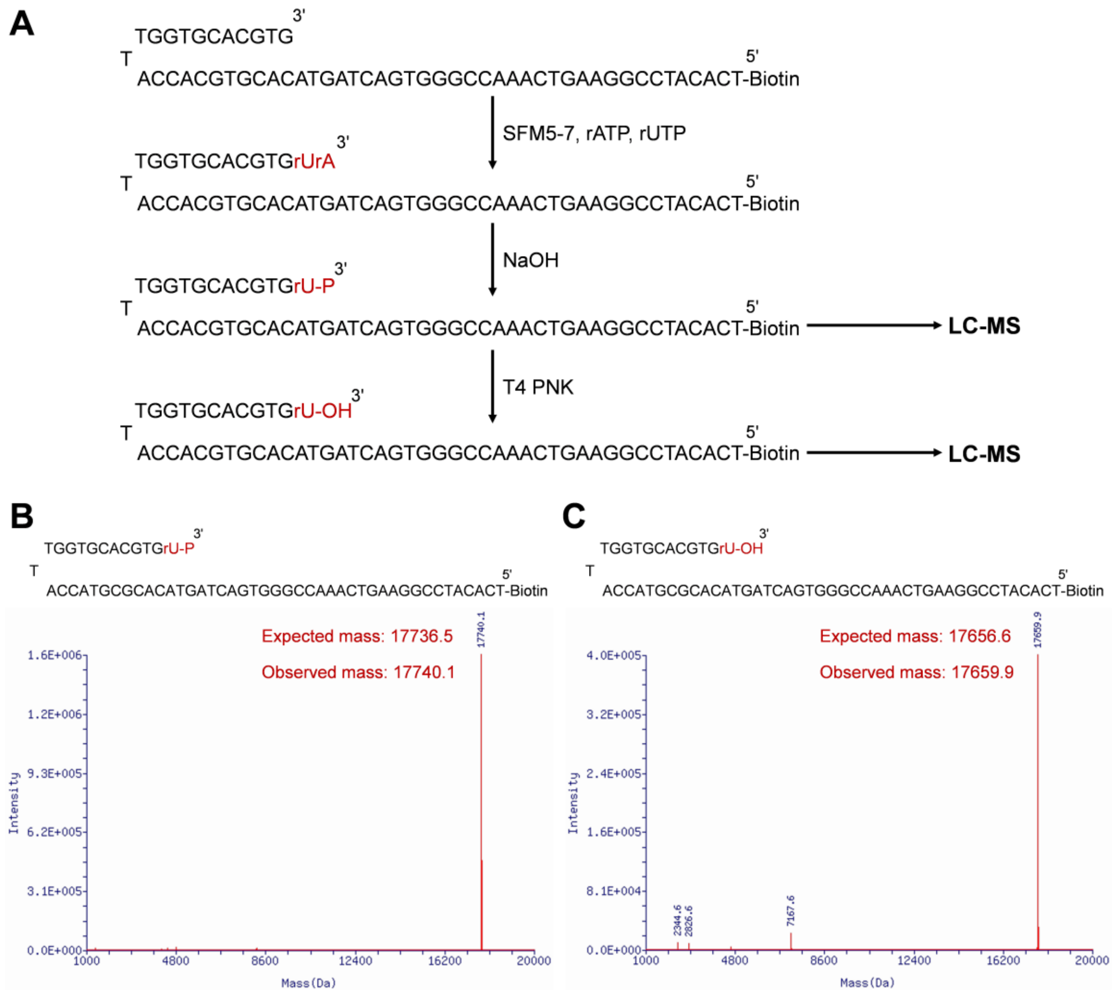

Figure S20. LC-MS assay for validating the strategy of using T4 PNK to regenerate the self-priming hairpin DNA template. (**A**) Scheme of the experiment. Self-priming hairpin DNA template B-T-55U was extended with rATP, rUTP and SFM5-7 and the product was incubated with NaOH as described above. The NaOH cleavage product was incubated with T4 PNK or not, purified with a Zymo ssDNA/RNA Clean & Concentrator™ kit and then sent for analysis with LC-MS. (**B**) LC-MS spectrum of the self-priming hairpin DNA template produced from NaOH cleavage. (C) LC-MS spectrum of the self-priming hairpin DNA template produced from NaOH cleavage and treated with T4 PNK.

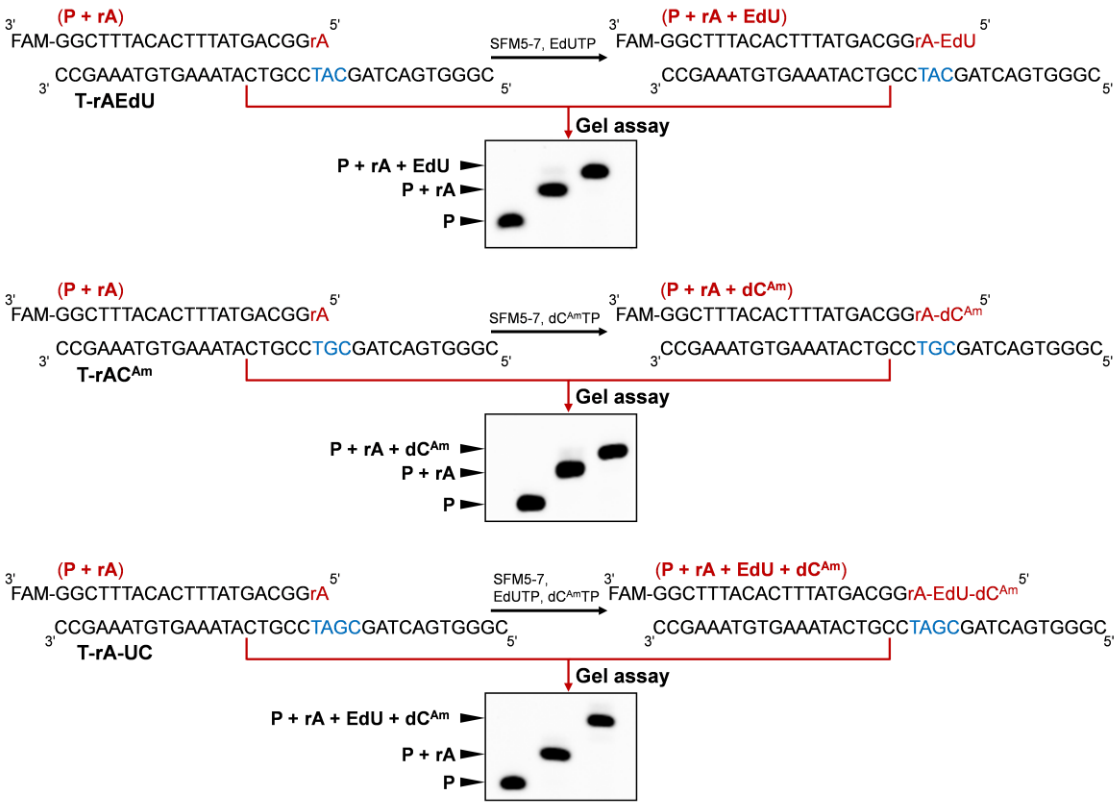

Figure S21. Assay for the incorporation of a single EdU or dC^{Am} nucleotide, or an EdU and a dC^{Am} nucleotides opposite the corresponding deoxyribonucleotide (s) in the template by SFM5-7. FAM-labeled primer FAM-P20 was annealed with template T-rAEdU, T-rAC^{Am} or T-rA-UC and extended with rATP by SFM5-7 to incorporate an rA nucleotide into its 3' end, and the product was purified as described in the materials and methods section. An EdU or dC^{Am} nucleotide was incorporated into the 3' end of the primer FAM-P20 harboring the 3'-end rA nucleotide by mixing 0.2 μM of the primer/template (T-rAEdU or T-rAC^{Am}) with 1 μM EdUTP or 1 μM dC^{Am}TP, and 1 μM SFM5-7 in 1× standard Taq buffer and incubating the mixture at 50 °C for 2 min. An EdU and a dC^{Am} nucleotides were incorporated into the 3' end of the primer FAM-P20 harboring the 3'-end rA nucleotide by mixing 0.2 μM of the primer/template (T-rA-UC) with 1 μM EdUTP, 1 μM dC^{Am}TP and 1 μM SFM5-7 in 1× standard Taq buffer and incubating the mixture at 50 °C for 2 min. The products were analyzed with 20% denaturing PAGE gels containing 8 M urea. P: FAM-labeled primer FAM-P20 (20 nt).

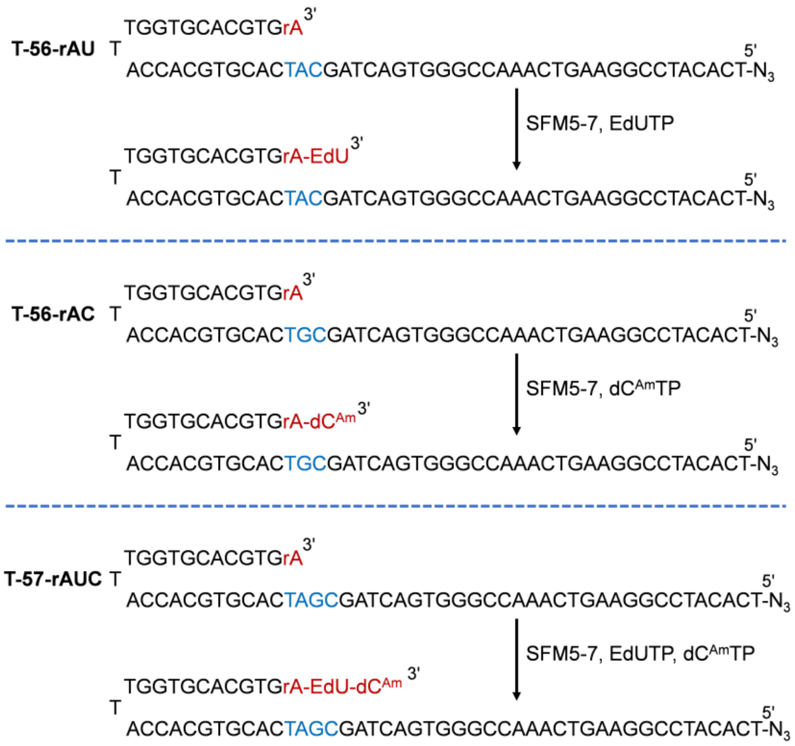

Figure S22. Design of the self-priming hairpin DNA templates for the production of 5'-labeled FANA or other XNA oligonucleotides. A self-priming hairpin DNA template was designed and synthesized to have different nucleotides at the positions opposite the positions where the first and second nucleotides will be incorporated, and different nucleotides at the positions opposite the positions where the second and third nucleotides will be incorporated when the primer is extended. For the dual labeling experiment, the nucleotides at the positions opposite the positions where the third and fourth nucleotides will be incorporated are also different. An rA nucleotide would be incorporated into the 3' end of 5'-azide-labeled self-priming hairpin DNA template T-56-rAU, T-56-rAC or T-57-rAUC, respectively, and the products would be purified. For the production of an FANA or another XNA oligonucleotide with a single-labeled 5' end, an EdU or dC^{Am} nucleotide would be incorporated into the 3' end of template T-56-rAU or T-56-rAC harboring the 3'-end rA nucleotide by mixing 0.2 μM of the template with 1 μM EdUTP or 1 μM dC^{Am}TP and 1 μM SFM5-7 in 1× standard Taq buffer and incubating the mixture at 50 °C for 2 min. For the production of an FANA or another XNA oligonucleotide with a dual-labeled 5' end, an EdU and a dC^{Am} nucleotides would be incorporated into the 3' end of template T-57-rAUC harboring the

3'-end rA nucleotide by mixing 0.2 μM of the template with 1 μM EdUTP, 1 μM $dC^{Am}TP$ and 1 μM SFM5-7 in 1× standard Taq buffer and incubating the mixture at 50 °C for 2 min.

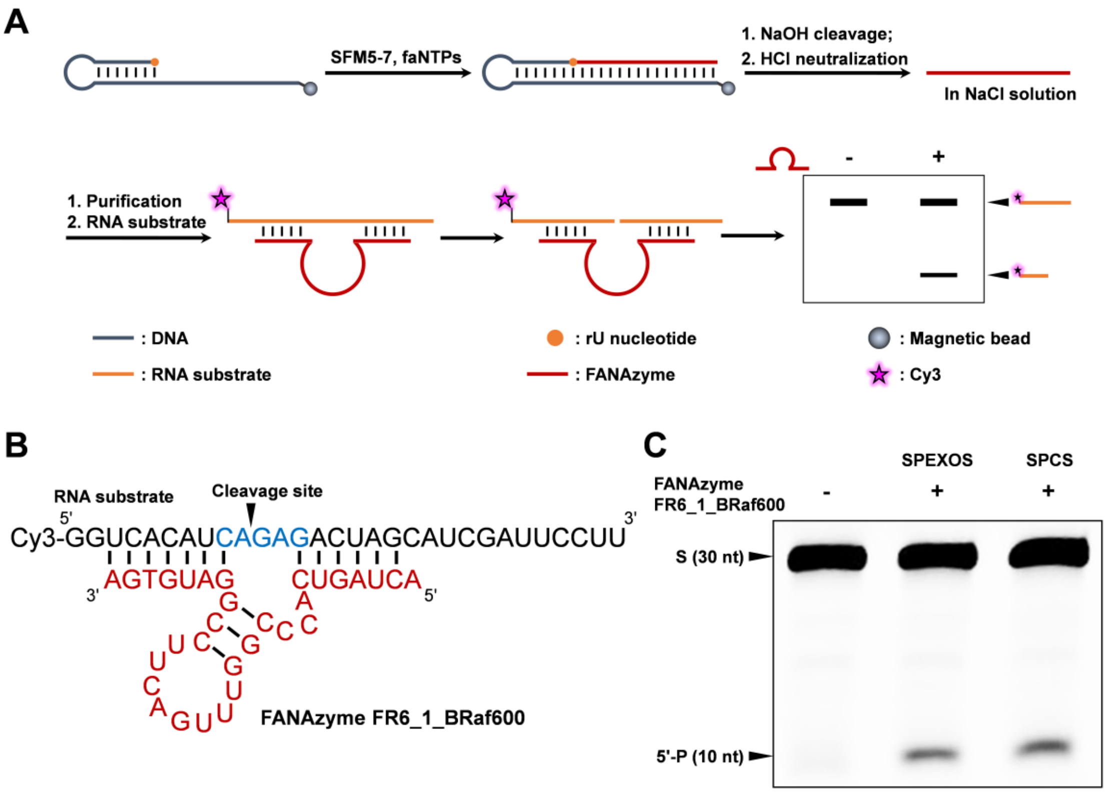

Figure S23. Production of a functional FANAzyme with the SPEXOS platform and verification of its activity. (**A**) Experimental scheme for the experiment. (**B**) The predicted secondary structure of the complex of FANAzyme FR6_1_BRaf600 and its RNA substrate, whose 5'-end is labeled with Cy3, with the cleavage site of the RNA substrate marked. (**C**) Verification of the activity of FANAzyme FR6_1_BRaf600 produced by the SPEXOS platform. 10 μM of FR6_1_BRaf600 produced by solid-phase chemical synthesis (SPCS) or the SPEXOS platform (SPEXOS) was mixed with 2 μM of the RNA substrate and 1 mM $MgCl_2$ in 10 mM Tris-HCl (pH 7.5), and incubated at 37 °C for 15 h to cleave the RNA substrate. The cleavage products were analyzed by denaturing PAGE. S: RNA substrate Cy3-R30; 5'-P: the upstream RNA cleavage product with the 5'-end Cy3 label.

Reference

1. Chen T, Hongdilokkul N, Liu Z *et al*. Evolution of thermophilic DNA polymerases for the recognition and amplification of C2'-modified DNA. *Nat Chem* 2016; **8**: 556-562.
2. Chen T, Romesberg FE. Enzymatic synthesis, amplification, and application of DNA with a functionalized backbone. *Angew Chem Int Ed* 2017; **56**: 14046-14051.
3. Qin Y, Ma X, Tao R *et al*. Synthesis, reverse transcription, replication, and inter-transcription of 2'-modified nucleic acids with evolved thermophilic polymerases: Efforts toward multidimensional expansion of the central dogma. *ACS Synth Biol* 2023; **12**: 2616-2631.
4. Medina E, Yik EJ, Herdewijn P *et al*. Functional comparison of laboratory-evolved XNA polymerases for synthetic biology. *ACS Synth Biol* 2021; **10**: 1429-1437.
5. Wu KB, Skrodzki CJ, Su Q *et al*. "Click handle"-modified 2'-deoxy-2'-fluoroarabino nucleic acid as a synthetic genetic polymer capable of post-polymerization functionalization. *Chem Sci* 2022; **13**: 6873-6881.
6. Pinheiro VB, Taylor AI, Cozens C *et al*. Synthetic genetic polymers capable of heredity and evolution. *Science* 2012; **336**: 341-344.
7. Liu C, Cozens C, Jaziri F *et al*. Phosphonomethyl oligonucleotides as backbone-modified artificial genetic polymers. *J Am Chem Soc* 2018; **140**: 6690-6699.
8. Cozens C, Pinheiro VB, Vaisman A *et al*. A short adaptive path from DNA to RNA polymerases. *Proc Natl Acad Sci USA* 2012; **109**: 8067-8072.
9. Li Q, Maola VA, Chim N *et al*. Synthesis and polymerase recognition of threose nucleic acid triphosphates equipped with diverse chemical functionalities. *J Am Chem Soc* 2021; **143**: 17761-17768.
10. Nikoomanzar A, Vallejo D, Yik EJ *et al*. Programmed allelic mutagenesis of a DNA polymerase with single amino acid resolution. *ACS Synth Biol* 2020; **9**: 1873-1881.
11. Torres LL, Pinheiro VB. Xenobiotic Nucleic Acid (XNA) Synthesis by phi29 DNA polymerase. *Current Protocols in Chemical Biology* 2018; **10**: e41.
